# Species delimitation and taxonomic revision of the *Rhamnella franguloides* complex (Rhamnaceae)

**DOI:** 10.3897/phytokeys.279.204054

**Published:** 2026-09-18

**Authors:** Qianna An, Zhiqiang Lu, Xiaolong Zhou

**Affiliations:** 1 College of Ecology and Environment, Xinjiang University, Urumqi 830046, China College of Ecology and Environment, Xinjiang University Urumqi China; 2 Xishuangbanna Tropical Botanical Garden of Chinese Academy of Sciences, Mengla 666303, China Xishuangbanna Tropical Botanical Garden of Chinese Academy of Sciences Mengla China

**Keywords:** Geographic distribution, ITS sequence, morphological cluster, *Rhamnella franguloides* complex, synonym

## Abstract

Integrative species delimitation requires at least two independent lines of evidence, such as morphological and molecular data. *Rhamnella franguloides* (including its synonyms and the varieties *inaequilatera* and *franguloides*) and *R. caudata* constitute a complex with poorly defined morphological boundaries. Here, we investigated species boundaries within this complex. Consistent with previous treatments, we recognized *Microrhamnus taquetii* as a synonym of *Euonymus alatus*, rather than of *R. franguloides*. Population-level morphological clustering and comparison based on all available specimens, together with geographic distributions, supported the recognition of at least three distinct clusters corresponding to three species in this complex: *R. franguloides*, *R. congesta* (S.Moore) Z.Qiang Lu & X.L.Zhou, and *R. caudata*. In addition, the two varieties of *R. franguloides* are distinguished by markedly different petiole lengths. These three species and two varieties are completely or largely allopatric, and their newly defined morphological boundaries and corresponding taxonomic treatments differ substantially from those proposed previously. Sequence variation in the internal transcribed spacer (ITS) region strongly supported the genetic distinction among the three species. Phylogenetic analysis based on ITS sequences indicated that the well-supported *R. congesta* clade was sister to another well-supported clade comprising *R. franguloides* and *R. caudata*. This study highlights the importance of integrating multiple lines of evidence in species delimitation and taxonomic revision.

## Introduction

Species delimitation is a fundamental task in biodiversity research (Wiens 2007). Over the past several decades, the proliferation of species concepts has underscored the limitations of relying on a single criterion to delimit species (Hey 2006; Liu 2016; Zachos 2016). A growing body of evidence indicates that single-evidence approaches can introduce systematic bias or lead to errors in species delimitation (e.g., Sukumaran and Knowles 2017; Lu et al. 2024). Different species’ concepts emphasize distinct attributes, such as morphological diagnosability, reproductive isolation, phylogenetic relationships, and ecological divergence, yet any single concept may be inadequate when applied to cryptic species, asexual lineages, recently diverged taxa, or sympatrically distributed lineages (de Queiroz 2007; Liu 2016; Zachos 2016). Consequently, an integrative framework that combines multiple lines of evidence is increasingly recognized as essential for robust species delimitation in taxonomic, ecological, and conservation research (de Queiroz 2007; Wiens 2007; Padial et al. 2010; Liu 2016).

Among the available criteria, morphological diagnosability remains one of the most practical and widely applied criteria for species delimitation (Dayrat 2005; Sukumaran and Knowles 2017; Lu et al. 2021). However, its reliable application requires population-level statistical comparisons, as analyses based on few specimens may confound intraspecific variation with interspecific differences (e.g., Lu et al. 2024). Morphological characters often vary across ontogenetic stages and during organ development (Crisp and Weston 1993; Espinosa et al. 2021); failing to account for such variation can result in substantial errors in estimates of species diversity (Buitrago Aristizábal et al. 2020). Moreover, hybrids frequently exhibit traits that differ from those of their parental species, and environmental heterogeneity can generate extensive phenotypic variation (López-Caamal et al. 2013), potentially leading to the erroneous recognition of such variants as independently evolving lineages (e.g., Zalewska-Gałosz et al. 2018; Lu et al. 2024). Therefore, species delimitation based on morphology should use specimens at comparable, morphologically stable developmental stages and be further validated using other independent lines of evidence.

An independent evolutionary lineage is a population or group of populations that evolves separately from other such lineages and maintains its evolutionary distinctiveness over time (de Queiroz 2007). Under an integrative species-delimitation framework, the recognition of a species should be supported by at least two independent lines of evidence, such as morphological differentiation, molecular divergence, reproductive isolation, or ecological differentiation (de Queiroz 2007; Padial et al. 2010; Liu 2016; Sukumaran and Knowles 2017; Lu et al. 2024). DNA barcoding, which uses short standardized DNA regions, provides a practical tool for identifying genetic distinction and distinguishing evolutionary lineages, particularly at the species level (Hebert et al. 2003). Numerous studies of plants have successfully used DNA barcodes to uncover cryptic or previously unrecognized species and to reinstate species previously placed in synonymy (Hu et al. 2015; Su et al. 2015; Lu et al. 2024; Liu et al. 2026). Thus, DNA barcodes can provide independent molecular evidence for evaluating lineages initially identified based on morphology, while geographic distribution patterns can provide additional corroborating evidence (e.g., Lu et al. 2021, 2024).

**Figure 1.**
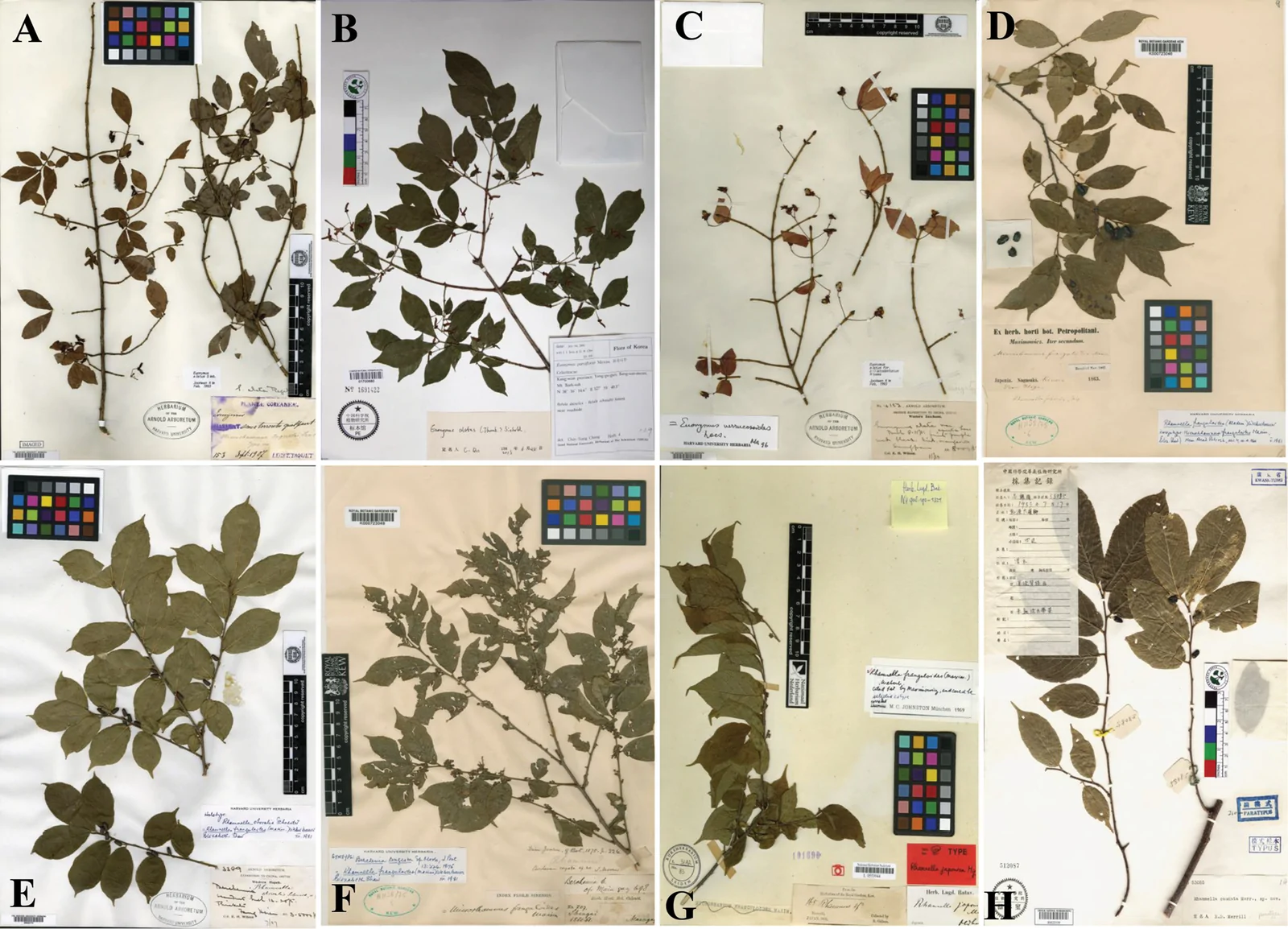
Representative specimens of the *R. franguloides* complex and *Euonymus alatus*. **A**. Type specimen of *Microrhamnus taquetii* (barcode: A00003587) from Korea; an annotation by J.-H. Kim (February 1993) on the type sheet identifies this specimen as *E. alatus*, and this treatment is also supported by Ma (2001); **B**. Representative specimen of *Euonymus alatus* (barcode: PE01700680) from Korea; **C**. Type specimen of Euonymus alatus var. apertus Loes. (barcode: A00003586) from China; **D**. Syntype of Rhamnella franguloides var. franguloides (barcode: K000723046); **E**. Holotype of *Rhamnella obovalis* (barcode: A00032772); **F**. Syntype of *Berchemia congesta* S.Moore (barcode: K000723049); **G**. Lectotype of *Rhamnella japonica* Miq. (barcode: L0553944); **H**. Paratype of *Rhamnella caudata* Merr. (barcode: PE00023559). Full specimen information and database sources are listed in table SS1. **A**. Shows opposite leaves and branches, branches bearing at least two rows of corky wings, subquadrangular branchlets, and rounded winter buds with irregularly and sharply denticulate bud-scale margins. These are key characters of *Euonymus alatus*, consistent with (**B, C**). Resembled morphology is shown between (**D, G**), and between (**E, F**) or (**H**).

**Figure 2.**
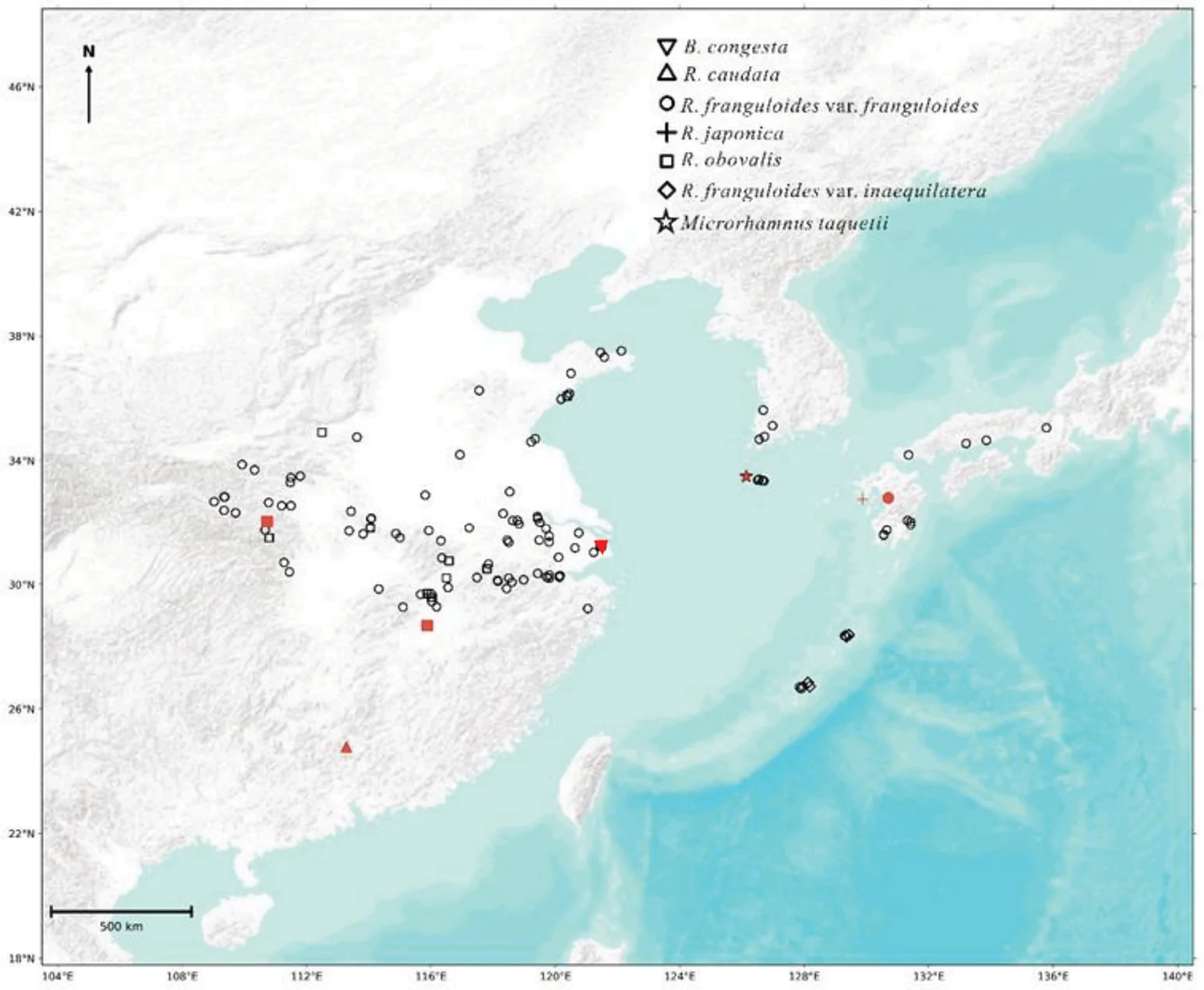
Geographical distribution of the *R. franguloides* complex. Different symbols represent the different species or variety names and synonyms. Red colors mark the type specimens.

The genus *Rhamnella* Miq. (Rhamnaceae) comprises deciduous shrubs or small trees and evergreen climbing shrubs and includes 12 accepted species distributed mainly in East and Southeast Asia (Richardson et al. 2000; Chen and Schirarend 2007; Hauenschild et al. 2016; Lu and Sun 2019, 2020; POWO 2026). Morphological distinctions among Rhamnaceae taxa are often subtle and easily overlooked (Richardson et al. 2000; Chen and Schirarend 2007; Hauenschild et al. 2016). Recent studies suggest that the diagnostic characters used to distinguish *Rhamnella* species include leaf habit (deciduous or evergreen), pubescence, petiole length, leaf length-to-width ratio, and the shape and size of dried fruits (Lu and Sun 2019, 2020). Nevertheless, the morphological boundaries of several species remain unclear (Chen and Schirarend 2007). For instance, the type species, *R. franguloides* (≡*Microrhamnus franguloides* Maxim.), has numerous synonyms and its type specimen, collected in Japan, bears glabrous petioles and longer leaf apices, whereas the type specimen of *R. obovalis* C.K.Schneid. (a synonym of *R. franguloides*), collected in China, bears densely pubescent petioles and shorter leaf apices (Fig. 2; Ohwi 1951). Furthermore, the type specimens of *Berchemia congesta* S.Moore from China and *Rhamnella japonica* Miq. from Japan—both names currently treated as synonyms of *R. franguloides*—closely match the typical morphology of *R. obovalis* and *R. franguloides*, respectively (Figs 1, 2). These findings raise the question of whether *Berchemia congesta* or *R. obovalis* should be reinstated as a distinct species.

It should also be noted that *Microrhamnus taquetii* H.Lév., a name currently treated as a synonym of *R. franguloides*, was previously placed in synonymy with *E. alatus* by Kim (1993) and Ma (2001). This type material is listed here (Fig. 1A). An annotation by Kim (February 1993) on the type sheet identifies this specimen as *E. alatus* (Fig. 1A). However, Chen and Schirarend (2007) argued that *M. taquetii* should instead be treated as a synonym of *R. franguloides*. Moreover, *R. franguloides* comprises two varieties, var. *franguloides* and var. *inaequilatera* (Ohwi) Hatus. (Ohwi 1951; Iwatsuki et al. 1999), which are distributed in central, eastern, and southeastern China, the Liuchiu (Ryukyu) Islands, Korea, and Japan (Fig. 2). The two varieties are distinguished by petiole length (3–8 mm vs. 10–15 mm), the spacing of crenate-serrulate teeth along the leaf margins (narrowly vs. widely spaced), leaf-base symmetry (equal-sided vs. partly unequal-sided), and fruit striation (absent vs. present), respectively (Ohwi 1951). However, examination of some specimens indicates that most of these characters are variable or non-diagnostic: petioles as short as 7.8 mm occur in var. *inaequilatera* (e.g., K004542032); var. *franguloides* also has 2-locular pyrenes (Chen and Schirarend 2007) and unequal-sided leaf bases (e.g., E00275330, S-G-5219). Thus, these observations raise two additional questions about taxonomic boundaries and treatments within *R. franguloides*: 1) whether *M. taquetii* should be treated as a synonym of *R. franguloides* or *E. alatus*; 2) whether the two varieties can be reliably distinguished morphologically.

**Figure 3.**
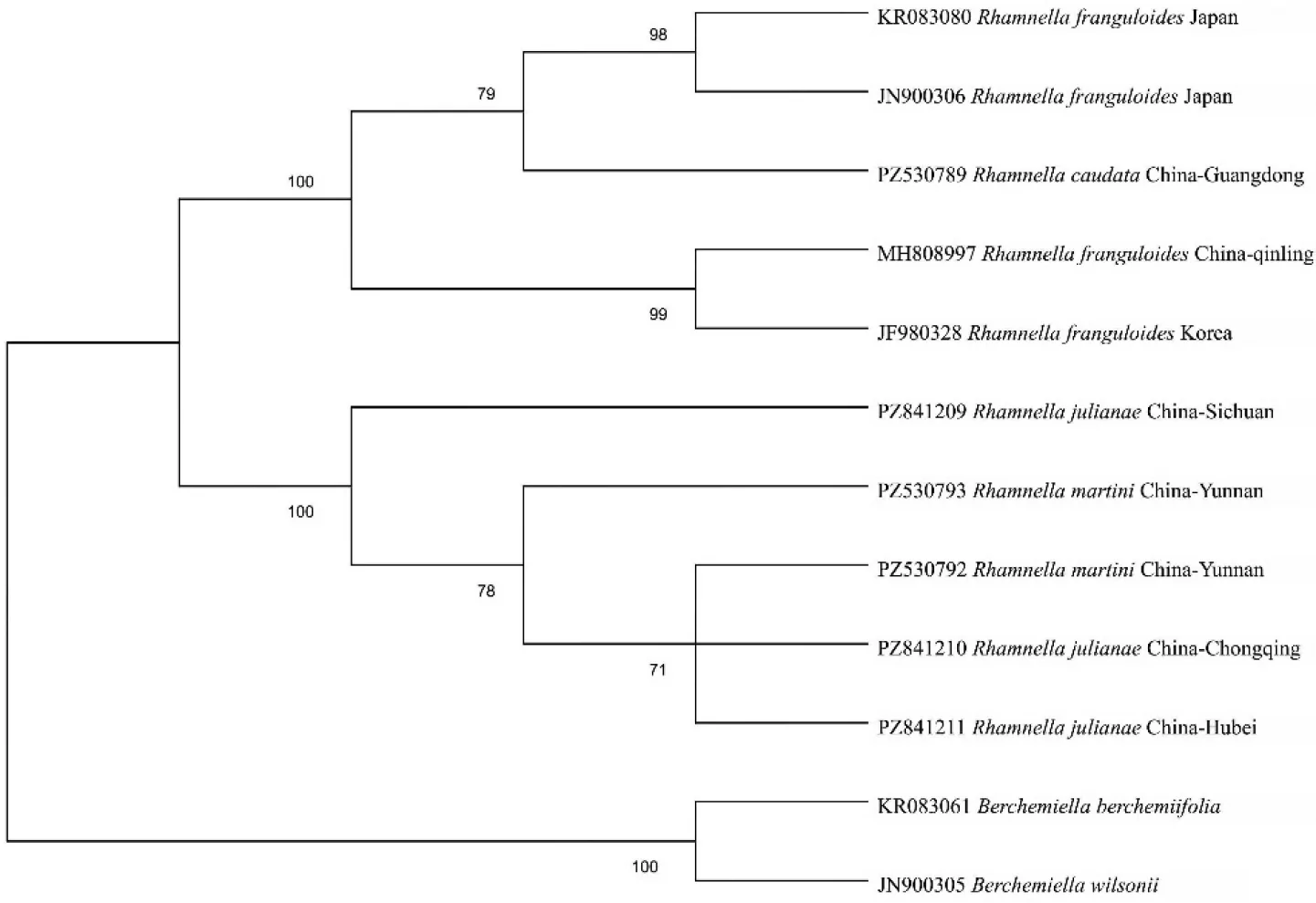
The reciprocal monophyly between the *R. franguloides* complex and the *R. julianae* or *R. martini* based on phylogenetic analysis of nuclear ITS sequence variations using the Maximum Likelihood method. Bootstrap support values above 50% are shown. *Berchemiella berchemiifolia* and *B. wilsonii* are used as the outgroup.

The type specimen of *Rhamnella caudata* Merr. from Guangdong, China, resembles that of *R. franguloides* in leaf shape, petiole length, and fruit characters based on their descriptions, and the geographic distribution of *R. caudata* is relatively close to that of Chinese populations of *R. franguloides* (Figs 1H, 2; Ohwi 1951; Chen and Schirarend 2007). Two other species, *R. julianae* C.K.Schneid and *R. martini* (H.Lév.) C.K.Schneid, also occur in close geographic proximity to Chinese populations of *R. franguloides* (Chen and Schirarend 2007). However, molecular evidence distinguishes these two species from both *R. caudata* and *R. franguloides* (Lu and Sun 2020; Fig. 3). Moreover, all other deciduous congeners are distinctly different from both *R. caudata* and *R. franguloides* in morphology and geographic distribution (Chen and Schirarend 2007), whereas *R. caudata* is phylogenetically nested within *R. franguloides* (Fig. 3). Therefore, *R. franguloides* and *R. caudata* together constitute a complex with poorly defined morphological and genetic boundaries. This raises the final taxonomic question of whether *R. caudata* should be treated as a synonym of *R. franguloides*.

In this study, we found that *M. taquetii* differs from *R. franguloides* but matches *E. alatus*, supporting Kim’s and Ma’s taxonomic treatments (Kim 1993; Ma 2001). To address the three unresolved taxonomic questions in the *R. franguloides* complex, we first conducted statistical analyses of morphological variation using all available specimens across the geographic range of the species complex to identify distinct morphological clusters. Second, we mapped the geographic distributions of all identified clusters. Finally, we applied DNA barcoding to representatives of each cluster to clarify their evolutionary relationships and genetic divergences. By integrating morphological, molecular, and geographic evidence, we recognize at least three species (*R. franguloides*, *R. congesta*, and *R. caudata*) rather than two within this complex. We also redefine the morphological boundaries between *R. franguloides* and *R. caudata* and between the two varieties (var. *inaequilatera* and var. *franguloides*), treat *R. congesta* as a distinct species, with *R. obovalis* as its synonym; and clarify the geographic distributions of the newly circumscribed *R. franguloides*, its two varieties, and *R. congesta*.

## Material and methods

### Search for other species with similar specimens to *Microrhamnus taquetii* in Korea

We recorded morphological characters from the type specimen of *Microrhamnus taquetii* (i.e., A00003587) (Fig. 1A; Suppl. material 1: table SS1). Using these characters, we then searched for additional specimens exhibiting similar morphology through the online databases of the Global Biodiversity Information Facility (GBIF: https://www.gbif.org/) and the Chinese Virtual Herbarium (CVH: http://www.cvh.ac.cn/en). Subsequently, we compared these specimens with those of *M. taquetii* to determine the genus and species to which they should be assigned.

### Sample collection

We reviewed the specimen records of the *R. franguloides* complex available through CVH and GBIF (Suppl. material 1: tables S1–S3). Based on these databases and herbaria, we compiled locality data and generated a distribution map (Fig. 2). When labels provided precise coordinates, these were transcribed directly; otherwise, coordinates were georeferenced from locality descriptions using online mapping resources (Suppl. material 1: table SS2). We subsequently conducted field surveys to collect fresh leaves and voucher specimens from representative populations for molecular and morphological analyses, respectively. The freshly collected leaves were immediately desiccated in silica gel. For each population, we obtained at least one representative voucher specimen for morphological analysis and sampled between two and 13 individuals for DNA sequencing (Table 1). In addition, we collected leaf samples from *R. julianae* and *R. martini* for sequencing. Geographic coordinates of the newly sampled populations were recorded in the field using a handheld global positioning system (GPS) device. All voucher specimens were deposited in the Herbarium of Xinjiang University (**XJU**).

**Table 1.** The newly collected samples for morphological and molecular analyses.

**Species name**	**Collector**	**Voucher number**	**Collection site**	**Elevation (m)**	**GPS coordinates**	**Herbarium**
*R. franguloides*	X.L. Zhou	2025YJ01–2025YJ04	Yuhuangjian, Henan, China	866	33.73°N, 110.81°E	XJU
*R. franguloides*	X.L. Zhou	2025PL01–2025PL02	Pingli, Shaanxi, China	651	32.39°N, 109.21°E	XJU
*R. franguloides*	X.L. Zhou	2025XD01	Xiangxidong, Shaanxi, China	454	32.66°N, 109.03°E	XJU
*R. franguloides*	X.L. Zhou	2025NJ01–2025NJ02	Nanjing, Jiangsu, China	449	32.06°N, 118.22°E	XJU
*R. franguloides*	X.L. Zhou	2025ZC01–2025ZC02	Zoucheng, Shandong, China	97	35.09°N, 116.44°E	XJU
*R. franguloides*	X.L. Zhou	2025FX01–2025FX03	Fangxian, Hubei, China	800	32.03°N, 110.44°E	XJU
*R. franguloides*	X.L. Zhou	2025FS01–2025FS02	Fanguangsi, Zhejiang, China	467	29.27°N, 121.06°E	XJU
*R. caudata*	X.L. Zhou	2025RY01–2025RY13	Ruyuan, Guangdong, China	367	25.34°N, 113.28°E	XJU
*R. martini*	X.L. Zhou	2025XS01–2025XS02	Xishan, Yunnan, China	2200	24.58°N, 102.38°E	XJU
*R. julianae*	X.L. Zhou	2026WX01	Wuxi, Chongqing, China	1470	31.42°N, 109.35°E	XJU
*R. julianae*	X.L. Zhou	2026MY01	Muyu, Hubei, China	1600	31.20°N, 110.30°E	XJU
*R. julianae*	X.L. Zhou	2026WC01	Wenchuan, Sichuan, China	1510	31.22°N, 103.40°E	XJU

### Morphological analyses at the population level

We first examined morphological variation within the *R. franguloides* species complex. Morphological data were compiled from specimens accessed through GBIF and CVH following the methods of Lu et al. (2024). All quantitative traits were measured using ImageJ v. 1.49v, whereas qualitative traits were scored based on direct observations of the specimens. Newly collected and herbarium specimens were measured and compared within and among populations. At a larger scale, the specimens were divided into four geographic regions: China, South Korea, Japan, and the Liuchiu (Ryukyu) Islands (Figs 1, 2; Table 1), and morphological variation was further compared within and among regions. This approach enabled us to identify morphological variation both within and among populations or geographic regions. Morphological traits of leaves and branchlets were measured following the standardized protocols presented in Suppl. material 1: fig. S1, Tables 2, 3. The measured specimens of the *R. franguloides* complex covered its entire geographic range (Fig. 2). The type specimens of all synonyms were also examined (Fig. 1). Specifically, we measured seven specimens that were consistent with the protologue of R. franguloides var. inaequilatera (Ohwi 1951). To ensure consistency across the morphological dataset from all examined specimens, we selected seven quantitative and two qualitative traits of leaves and branchlets that exhibited clear differentiation between populations, geographic regions or taxa for the first morphological clustering analysis (Table 2). For example, lenticel density on old branchlet showed clear differentiation between populations, geographic regions or taxa, but we only obtained this dataset from 181 individuals in this complex, and all such traits were reflected by morphological comparison (Table 4). Our observations indicated that petiole length was relatively stable in leaves from the middle portions of branchlets throughout development. Therefore, only leaves from these portions were measured. Finally, a total of 412 specimens (388 identified as *R. franguloides*, including its varieties and synonyms, and 24 identified as *R. caudata*) were included in the first morphological clustering analysis, without missing data.

**Table 2.** Morphological characters used in the first PCA plotting.

**Code**	**Morphological characters**	**Unit**
a	Leaf length	Cm
b	Leaf width	Cm
c	Leaf length/width ratio	–
d	Mean petiole length	Cm
e	Leaf apex length	Cm
f	Leaf lateral vein number per side	–
g	a/f	–
h	Petiole pubescence, glabrous or nearly glabrous	1=Glabrous or nearly glabrous; 2=pubescent;
i	Annual branchlet pubescence, glabrous or nearly glabrous	1 = Glabrous or nearly glabrous; 2 = pubescent;

Note. Seven quantitative and two qualitative traits from leaves and branchlets were used for clustering analysis including all specimens.

**Table 3.** Morphological characters used in the second PCA plotting.

**Code**	**Morphological characters**	**Unit**
a	Leaf length	Cm
b	Leaf width	Cm
c	Leaf length/width ratio	–
d	Mean petiole length	Cm
e	Leaf apex length	Cm
f	Leaf lateral vein number per side	–
g	a/f	–
h	Petiole pubescence, glabrous or nearly glabrous	1=Glabrous or nearly glabrous; 2=pubescent;
i	Annual branchlet pubescence, glabrous or nearly glabrous	1=Glabrous or nearly glabrous; 2=pubescent;
j	Fruit height	Cm
k	Fruit width	Cm
l	Mean fruit pedicel length	Cm
m	Mean fruit length/width ratio	–
n	j × k	cm^2^
o	Mean petiole length /mean fruit pedicel length	–

Note. Thirteen quantitative and two qualitative traits were used for clustering analysis based on specimens with stable fruit size.

**Table 4.** Morphological comparison of three identified clusters and two varieties based on all measured specimens in the complex.

**Traits**	**Cluster A (*R. congesta*)**	**Cluster B (*R. caudata*)**	**Cluster C (var*. franguloides*)**	**Cluster C (var*. inaequilatera*)**
**LEAF**				
Shape and size	Leaf blade subelliptic, obovate, obovate-oblong, obovate-elliptic, oblong, or narrowly elliptic, 3.5–11.2 cm × 1.9–5.2 cm, length/width ratio 1.5–2.8; base partly unequal-sided vs. equal-sided, subcordate, rounded, subrounded, or cuneate; apex acute, short acuminate or caudate, rarely long caudate or long acuminate, apex length 0.22–0.90 cm	Leaf blade subelliptic, oblong, obovate-elliptic or obovate-oblong, 7.6–14.6 cm × 2.9–4.5 cm, length/width ratio 2.1–3.4; base partly unequal-sided vs. equal-sided, subcordate, rounded, subrounded, or cuneate; apex long caudate or long acuminate, rarely acute and short acuminate or caudate, apex length 0.87–2.40 cm	Leaf blade obovate-oblong, obovate-elliptic, oblong, or narrowly elliptic, rarely obovate, 6.8–14.3 cm × 2.4–5.9 cm, length/width ratio 2.3–3.3; base equal-sided or unequal-sided, rounded, subrounded, or cuneate; apex long caudate or long acuminate, rarely acute, apex length 0.83–1.86 cm	Leaf blade obovate-oblong, obovate-elliptic, oblong or long-oblong, 8.8–10.4 cm × 3.1–3.9 cm, length/width ratio 2.4–3.0; base equal-sided or unequal-sided, rounded, subrounded, or cuneate; apex abruptly acuminate, long acuminate or acute, apex length 0.78–1.48 cm
Leaf margin	Serrulate	Shallowly serrate	Serrulate or remotely crenate-serrulate	Remotely crenate-serrulate
Petiole length	Range 1.0–3.8 mm, mean 1.5–3.1 mm	Range 3.5–5.8 mm, mean 3.8–5.2 mm	Range 3.8–7.6 mm, mean 4.4–7.2 mm	Range 7.8–15.8 mm, mean 8.2–13.6 mm
Petiole glabrous or pubescent	Pubescent	Glabrous or nearly glabrous	Glabrous or nearly glabrous	Glabrous
lateral vein No. (per side)	5–9, rarely to 10–13	8–13, rarely to 7	6–13	5–10
Leaf length to lateral vein No. ratio	0.55–1.20	0.85–1.50	0.82–1.50	0.94–1.29
**BRANCHLET**				
Glabrous or pubescent for annual branchlet	Pubescent	Glabrous or nearly glabrous	Glabrous or nearly glabrous	Glabrous or nearly glabrous;
Lenticel No. (per cm^2^, old branchlet with 2 mm in diameter)	82% individuals 61–87, 18% individuals with 89–140	87–144	54-87	54-83
**FRUIT**				
Pyrene locule No.	1 or 2 locules	1 or 2 locules	1 or 2 locules	2 locules
Fruit surface (striate or not)	striate or not	striate or not	striate or not	striate
Dry fruit size	5.0–9.0 mm × 2.8–4.0 mm	9.0–10.9 mm × 3.6–4.4 mm	6.6–8.7 mm × 3.2–4.0 mm	5.5–7.2 mm × 2.9–3.8 mm
Fruit length to width ratio	1.50–2.68	2.05–2.76	1.75–2.50	2.13–2.27
Fruit pedicel length	2.0–4.0 mm, mean 2.5–3.5 mm	3.2–5.0 mm, mean 3.5–4.5 mm	3.0–5.0 mm, mean 3.2–4.3 mm	3.0–4.3 mm, mean 3.3–3.6 mm
Mean petiole length/mean pedicel length	0.50–1.03	0.95–1.34	1.38–2.18	2.31–2.49

Note: Excluding the type specimen of *R. japonica*, the morphological comparison of all above qualitative and quantitative traits has no any change. Lenticel density ranges of old branchlets are measured from 132 specimens of Cluster A, 15 specimens of Cluster B, and 34 specimens of Cluster C. Pyrene locule No. and fruit surface (striate or not) of var*. inaequilatera* follow Ohwi (1951).

Fruit size is an important taxonomic character for species delimitation in *Rhamnella* (Lu and Sun 2019, 2020). Field observations indicated that fruit size in *R. franguloides* stabilized by July. Therefore, specimens collected before July and those without fruits were excluded from the second morphological analysis. According to the above operation procedure, we then retained 372 specimens of *R. franguloides* complex with stable fruit size for the subsequent clustering analysis based on all 13 quantitative and two qualitative traits (Table 3), without missing data. This dataset included most of the key traits traditionally used to distinguish species in *Rhamnella*, such as pubescence, petiole length, leaf length-to-width ratio, and dried fruit shape and size (Chen and Schirarend 2007; Lu and Sun 2019, 2020). The dataset comprised 325 specimens from China previously identified as *Rhamnella franguloides* (including type specimens of *R. obovalis* and *B. congesta*), 20 *R. caudata* specimens from Guangdong, and 27 *R. franguloides* specimens from the Liuchiu (Ryukyu) Islands, Japan, and Korea.

The above two morphological clustering analyses were analyzed using principal component analysis (PCA). For each dataset, PCA was performed in R version 4.4.3 (R Core Team 2025) using the prcomp() function, with all variables centered and scaled to unit variance (scale. = TRUE) so that traits measured on different scales contributed equally. Because each dataset included continuous, binary and ratio variables, a Gower dissimilarity matrix was computed (Maechler et al. 2026). After generating the PCA plots, we compared the morphological differences among the identified clusters by PCA plotting.

### DNA extraction, PCR amplification and phylogenetic analysis

Total genomic DNA was extracted from silica-dried leaf material using a modified CTAB protocol (Doyle and Doyle 1990). Because the nuclear internal transcribed spacer (ITS) region has high discriminatory power at the species level in *Rhamnella* (Lu and Sun 2020), we preferentially selected this barcode for genetic delimitation. The ITS region was amplified using the primers ITS1a and ITS4a, according to the protocols described by Zhou and An (2025). PCR amplification was performed in 50 μL reactions containing 2 μL DNA template, 5 μL 10× PCR buffer, 0.8 μL dNTPs (2.5 mM), 2 μL of each primer (10 μM), 0.4–0.5 μL rTaq polymerase, and ddH_2_O. Amplification conditions were as follows: an initial denaturation at 94 °C for 4 min; 38 cycles of 94 °C (40 s), 60 °C (45 s), and 72 °C (2 min); and a final extension at 72 °C for 6 min. PCR products were verified by agarose gel electrophoresis, and products of sufficient quality were sequenced.

To illustrate that the complex is distinct from *R. julianae* and *R. martini*, we constructed a maximum likelihood (ML) tree based on the ITS sequences using MEGA12 with default parameters (Kumar et al. 2024). Bootstrap analysis was performed with 1,000 replicates. The tree was rooted with *Berchemiella berchemiifolia* and *B. wilsonii* (Fig. 3). The phylogenetic analysis supported the distinction of the complex from *R. julianae* and *R. martini* (Fig. 3). Subsequently, we constructed a second ML tree using the same method to present the evolutionary relationship among the identified clusters. The second tree was rooted with two individuals of *Rhamnella martini* (X.L. Zhou 2025XS01 and X.L. Zhou 2025XS02). *R. martini* was selected as the outgroup because it is phylogenetically distinct from the *R. franguloides* complex (Fig. 3; Lu and Sun 2020).

## Results

### *Microrhamnus taquetii* matching with *Euonymus alatus* (Thunb.) Siebold

Based on the type specimen of *Microrhamnus taquetii* (barcode No. A00003587), we extracted the following diagnostic morphological characteristics: a woody plant with simple, opposite leaves, ovate-elliptic to obovate, 2.0–5.5 cm long and 1–2.5 cm wide, with finely serrate margins, acuminate apices, and cuneate to obtuse bases; seven to eight pairs of lateral veins; petioles 1.0–3.0 mm long; branches opposite and distinctly bearing at least two rows of corky wings; branchlets subquadrangular; winter buds rounded, about 2 mm long, with the margins of the bud scales irregularly and sharply denticulate; fruits with at least 1–2 seeds, containing at least 1–2 locules, with a persistent sepal whose diameter is greater than that of the seed base (Fig. 1A). These characteristics, including opposite leaves and branches, branches with at least two rows of corky wings, subquadrangular branchlets, winter buds rounded and about 2 mm long, winter buds with bud scale margins irregularly and sharply denticulate, and fruit with a persistent sepal whose diameter is greater than the base of the seed, are typical characteristics of *Euonymus alatus* (a species of *Euonymus* in the family Celastraceae) (Fig. 1A–C). Therefore, *M. taquetii* should be excluded from the *R. franguloides* complex.

### Morphological clustering and comparison

**Figure 4.**
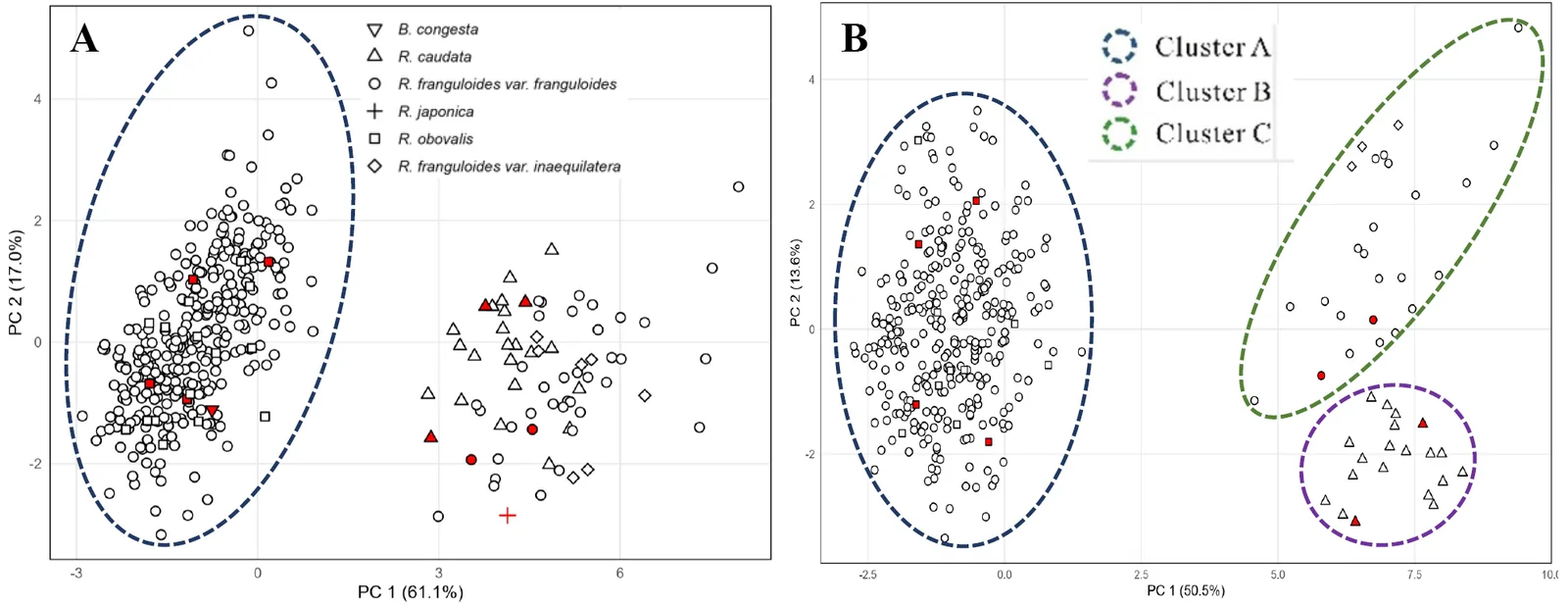
Principal component analyses (PCA) of morphological traits in the *Rhamnella franguloides* complex. **A**. The first PCA result using morphological datasets from vegetative organs (412 specimens, seven quantitative and two qualitative traits) recognizes the Cluster A; **B**. The second PCA result using morphological traits from vegetative and reproductive organs (372 fruiting specimens, 13 quantitative and two qualitative traits) recognizes three major clusters (A, B, and C). These three clusters are largely or completely allopatric (see Fig. 5). Dashed ellipses or circles with different colors (blue: Cluster A; purple: Cluster B; green: Cluster C) distinguish each of three clusters from another two. Symbols indicate the original identifications, the same as Fig. 2, and type specimens are filled in red color.

**Figure 5.**
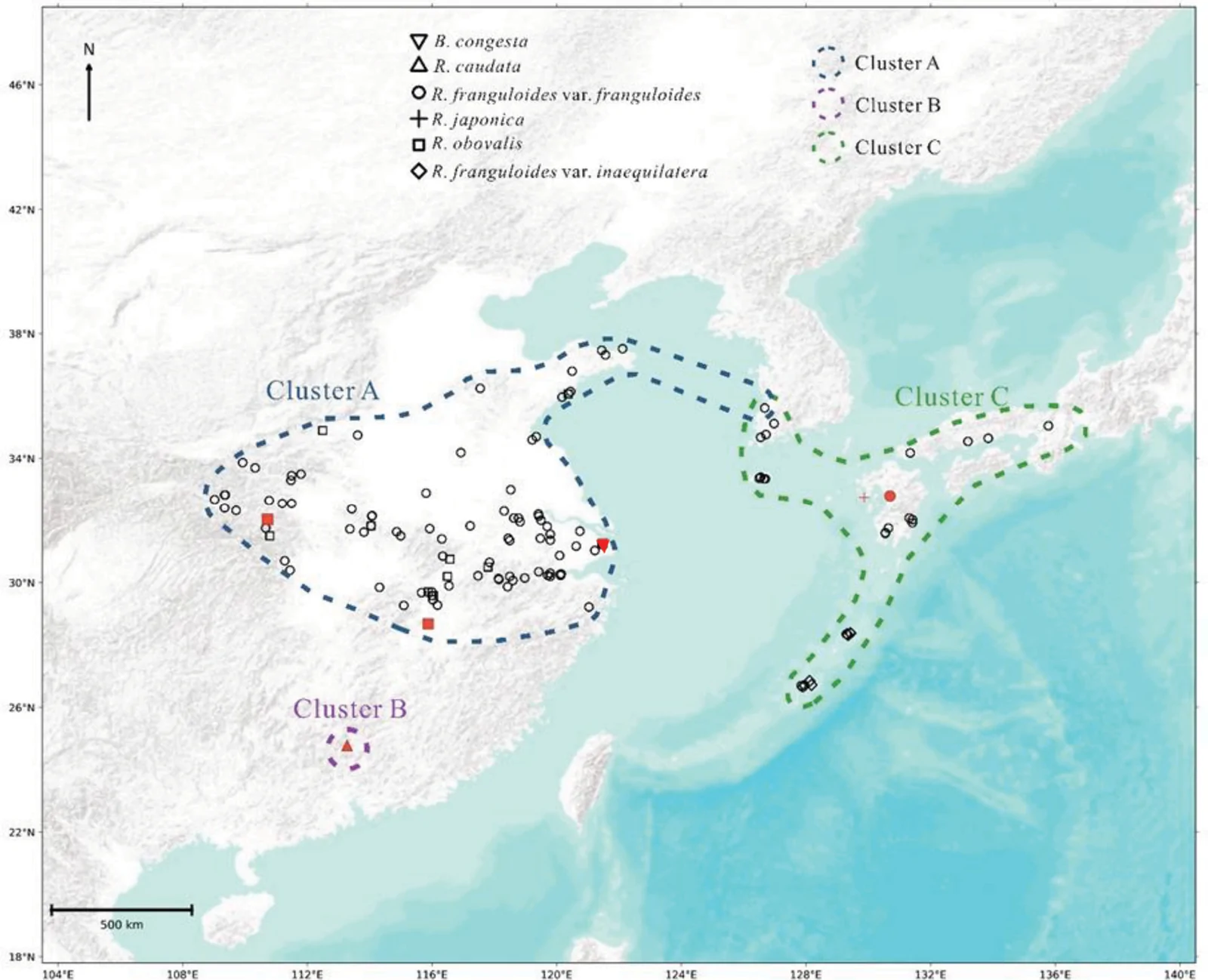
The newly outlined geographic distribution of each of three major clusters and two varieties of *R. franguloides* in the Cluster C based on Fig. 4 and Table 4. Dashed ellipses or circles with different colors (blue: Cluster A; purple: Cluster B; green: Cluster C) distinguish each of three clusters from another two. The dotted line within the Cluster C indicates geographic isolation between the two varieties identified in Table 4. Symbols indicate the original identifications, the same as Fig. 2, and type specimens are filled in red color.

Two groups were visually recovered from the first PCA result (Fig. 4A). One (Cluster A) comprised all specimens from China previously treated as *R. franguloides* (including the type specimens of *R. obovalis* and *Berchemia congesta*) plus one specimen from South Korea (Fig. 4A; Suppl. material 1: tables S1–S3), another comprised *R. caudata* together with all *R. franguloides* specimens from Japan, the Liuchiu (Ryukyu) Islands and the remaining specimens from South Korea. These two groups were mainly distinguishable by the absence or presence of petiole and annual branchlet pubescence, petiole length, and leaf apex length (Table 4). The second PCA result, based on 372 fruiting specimens, recovered three major clusters (Fig. 4B). Cluster A remained unchanged, whereas the second group in Fig. 4A was further divided into Cluster B (all individuals of *R. caudata*) and Cluster C (27 specimens from Japan, the Liuchiu (Ryukyu) Islands and South Korea). Cluster B and Cluster C were mainly distinguished by lenticel density and dry fruit size (Table 4). Notably, the type specimen of *R. japonica* (L0553944) lacked fruits and was therefore not included in the second PCA result. However, its specific lenticel count (74 per square meter) and extremely low number of lateral veins (6–7) for this specimen (L0553944) further supported its placement in Cluster C rather than Cluster B (Table 4). For the two varieties within Cluster C, morphological boundaries were redefined, with a distinct difference in leaf petiole length (Table 4). The geographic distributions of the three major clusters (A, B, and C) and two varieties (var. *inaequilatera* and var. *franguloides*) within Cluster C were outlined (Fig. 5).

### DNA sequence variation and molecular phylogenetics

This study generated new ITS sequences from 34 individuals, comprising 29 individuals of the *R. franguloides* complex, two individuals of *R. martini* (PZ530792–PZ530793), and three individuals of *R. julianae* (PZ841209–PZ841211) (Tables 5 and Suppl. material 1: table SS4). In addition, four sequences of the complex were retrieved from GenBank (Fig. 5; Table 5): MH808997 (China, Qinling Mountains) and JF980328 (South Korea) in Cluster A, and KR083080 and JN900306 (Japan or the Liuchiu (Ryukyu) Islands) in Cluster C. Thus, a total of 33 sequences were analyzed for the complex. Eight unique ITS sequence types (PZ530784–PZ530791) were detected among the newly generated sequences from the *R. franguloides* complex (Table 5 and Suppl. material 1: table SS4). The three major clusters showed significant differences from one another. Cluster A was distinguished from the other two clusters (B and C) by fixed mutation sites at positions 14 (C), 45 (G), 46 (A), 49 (T), and 176 (T), and Cluster B exhibited such diagnostic nucleotides (i.e., fixed mutation sites compared with the other two clusters) at positions 29 (T), 52 (T), 173 (C), 179 (T), and 416 (G). In addition, Cluster C had nine private sites when compared with either Cluster A or Cluster B, whereas Cluster B had ten private sites when compared with either Cluster A or Cluster C.

**Table 5.** Nuclear ITS sequence variations among three identified morphological clusters.

**Cluster (No. of individuals)**	**Geographic region**	**GenBank No**.	**Mutation site positions**																				
			**1**	**1**	**2**	**4**	**4**	**4**	**5**	**6**	**7**	**1**	**1**	**1**	**1**	**1**	**1**	**1**	**3**	**4**	**4**	**5**	**6**
			**2**	**4**	**9**	**5**	**6**	**9**	**2**	**1**	**0**	**0**	**1**	**2**	**5**	**7**	**7**	**7**	**5**	**1**	**8**	**9**	**1**
												**2**	**0**	**1**	**8**	**3**	**6**	**9**	**6**	**6**	**6**	**0**	**6**
Cluster A (1)	China	PZ530784	C	**C**	C	**G**	**A**	**T**	C	C	**G**	G	T	C	A	G	T	C	C	C	T	A	C
Cluster A (1)	China	PZ530785	C	**C**	C	**G**	**A**	**T**	C	C	**G**	G	Y	C	A	G	T	C	C	C	T	A	C
Cluster A (3)	China	PZ530787	C	**C**	C	**G**	**A**	**T**	C	C	**G**	A	C	C	A	G	T	C	C	C	T	A	Y
Cluster A (8)	China	PZ530788/ MH808997	C	**C**	C	**G**	**A**	**T**	C	C	**G**	R	Y	C	A	G	T	C	C	C	T	A	C
Cluster A (4)	China	PZ530786	T	**C**	C	**G**	**A**	**T**	C	C	**G**	G	T	C	A	G	T	C	C	C	T	A	C
Cluster A (1)	South Korea	JF980328	T	**C**	C	**G**	**A**	**T**	C	C	**G**	G	T	C	G	G	T	T	C	C	T	A	C
Cluster C (2)	Japan or Liuchiu (Ryukyu) Islands	KR083080/ JN900306	C	T	C	A	G	A	C	**T**	A	G	C	**T**	A	G	**C**	C	C	C	T	**G**	C
Cluster B (8)	China	PZ530789	C	T	**T**	A	G	A	**T**	C	A	G	C	C	A	**C**	T	C	C	**G**	**C**	A	C
Cluster B (3)	China	PZ530790	C	T	**T**	A	G	A	**T**	C	A	G	C	C	A	**C**	T	T	C	**G**	**C**	A	C
Cluster B (2)	China	PZ530791	C	T	**T**	A	G	A	**T**	C	A	G	C	C	A	**C**	T	Y	C	**G**	**C**	A	C

Note. Only ITS types in different regions or clusters are shown in this table; individual-level sequence information is given in Suppl. material 1: table SS4. The number of individuals sharing each type is indicated in parentheses. Except for MH808997, JF980328, KR083080, and JN900306, the remaining sequences were newly generated in this study. Variable positions are based on aligned sequences. Bold bases indicate positions where any one cluster differs from the other two clusters. Y = C/T, R = A/G.

**Figure 6.**
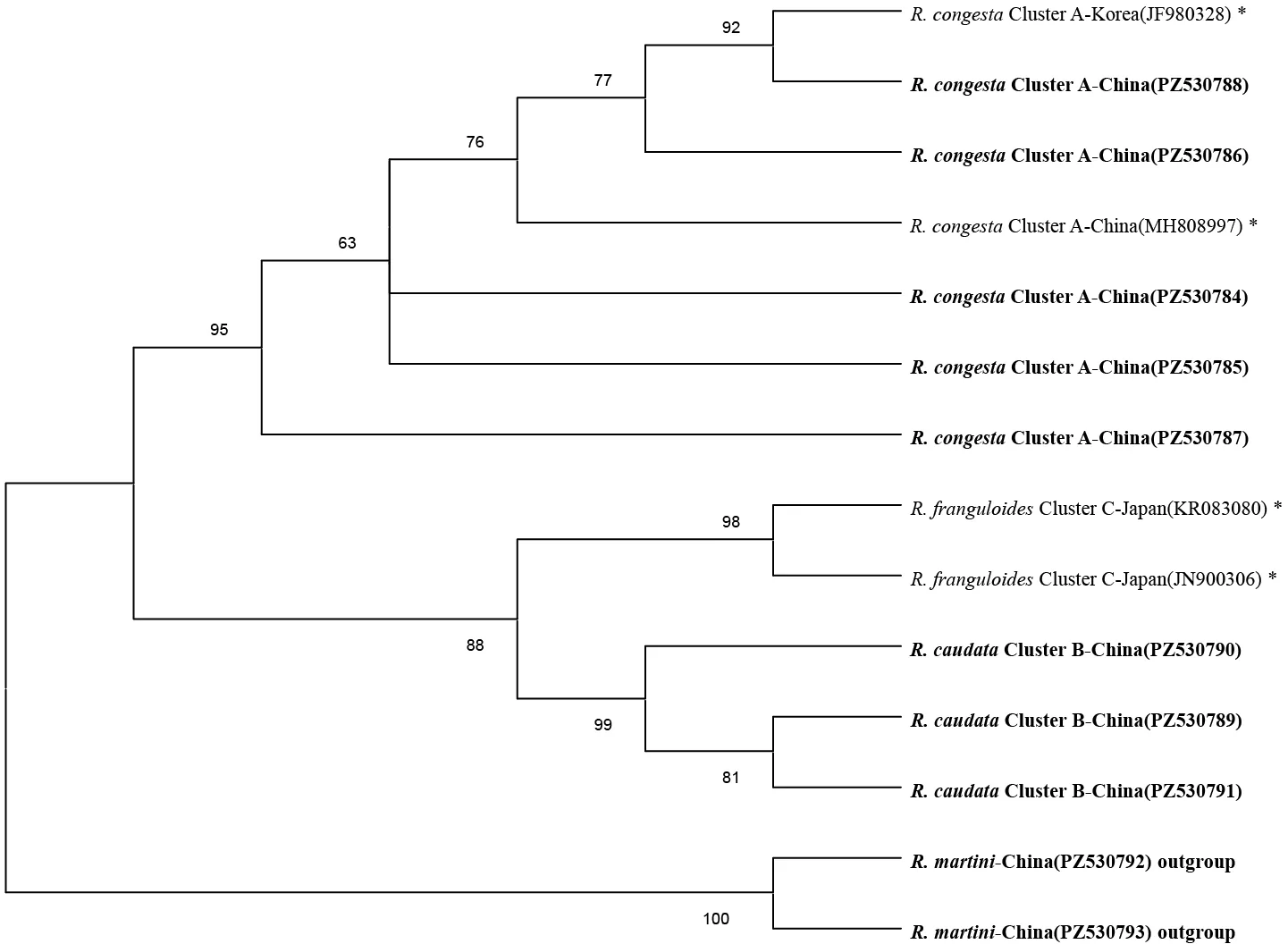
Phylogenetic relationship of the three major morphological clusters based on nuclear ITS sequence variations using the Maximum Likelihood method. Bootstrap support values above 50% are shown. Newly generated sequences are shown in bold; each downloaded sequence is marked with an asterisk. Two individuals of *R. martini* (X.L. Zhou 2025XS01, X.L. Zhou 2025XS02) were used as the outgroup. GenBank accession numbers were shown in parentheses.

Molecular phylogenetic analysis based on ITS sequence variation showed that the *R. franguloides* complex was reciprocally monophyletic with respect to *R. julianae* and *R. martini* (Fig. 3). Within the complex, Cluster A formed an independent clade that was sister to a well-supported clade containing Cluster B (*R. caudata*) and Cluster C (Fig. 6). Within the clade comprising Clusters B and C, the *R. caudata* (i.e., Cluster B) subclade comprised 13 individuals from Guangdong, China, whereas two individuals of Cluster C, representing both var. *inaequilatera* and var. *franguloides* or either of them, formed another subclade (Fig. 6; Table 1 and Suppl. material 1: table SS4). These two subclades were also well supported.

## Discussion

### *Microrhamnus taquetii* as a synonym *of Euonymus alatus*

Kim (1993) and Ma (2001) treated *Microrhamnus taquetii* (the type specimen voucher No.: *T. Taquet* 153) as a synonym of *Euonymus alatus*, consistent with the annotation by Kim (February 1993) on the type sheet (barcode No. A00003587; Fig. 1A). However, Chen and Schirarend (2007) argued that *M. taquetii* should instead be treated as a synonym of *R. franguloides* (Ma and Funston 2008; IPNI 2026; POWO 2026). Our direct re-examination of the type specimen (barcode No. A00003587) supports their treatments and the annotation by Kim (February 1993) on the type sheet, because the specimen exhibits a set of characters that are entirely inconsistent with *Rhamnella* but perfectly match those of *Euonymus alatus* (Celastraceae) (Fig. 1; Chen and Schirarend 2007; Ma and Funston 2008). These characters include opposite leaves and branches, branches bearing at least two rows of corky wings, subquadrangular branchlets, and rounded winter buds with irregularly and sharply denticulate bud-scale margins collectively, these are key characters for identifying *Euonymus alatus* (Ma and Funston 2008). In addition, they are fundamentally inconsistent with those of *R. franguloides* and its allies (Chen and Schirarend 2007). The type specimen of *M. taquetii* was collected in South Korea, where *E. alatus* also occurs (Fig. 1A–C). Therefore, the treatment of *M. taquetii* as a synonym of *R. franguloides* should be rejected, whereas its treatment as a synonym of *Euonymus alatus*, as proposed by Kim (1993), the annotation by Kim (February 1993) on the type sheet, and Ma (2001), is supported.

### Morphological delimitation and geographic distribution

Previous taxonomic treatments of the *R. franguloides* complex commonly recognized two species, one of which comprised two varieties: *R. franguloides* (var. *inaequilatera* and var. *franguloides*) and *R. caudata* (Ohwi 1951; Chen and Schirarend 2007; IPNI 2026; POWO 2026). Several names were also treated as synonyms of *R. franguloides* (Figs 1, 2), and no synonym was treated under *R. caudata* (Chen and Schirarend 2007). Population-level morphological clustering and comparison, together with geographic distributions, supported the recognition of at least three distinct clusters (A, B, and C) in this complex (Figs 4, 5; Table 4). Cluster A corresponds to the specimens previously identified as *R. franguloides* (including type specimens of *R. obovalis* and *B. congesta*) in China and one specimen from Korea, while Cluster B comprises only specimens of *R. caudata* from Guangdong, and Cluster C includes all specimens of the two varieties from Japan and the Liuchiu (Ryukyu) Islands and the remaining specimens from South Korea (Fig. 5; Table 4). These three clusters can be distinguished from one another based on the absence or presence of petiole and annual branchlet pubescence, lenticel density, petiole length, leaf apex length, and dry fruit size in the complex (Table 4). This finding differs markedly from the previous description and treatment (Chen and Schirarend 2007). Within Cluster C, var. *inaequilatera* was previously distinguished from var. *franguloides* by unequal-sided leaf bases, two–locular pyrenes, striate fruit surfaces, and leaf petiole length (10–15 mm) (Ohwi 1951). Among them, only leaf petiole length (3.8–7.6 mm vs. 7.8–15.8 mm) can distinguish the two varieties (Table 4), with new variable range different from the previous description (Ohwi 1951).

Allopatric speciation is considered a common model through which reproductive isolation develops (Coyne and Orr 2004). Although the development of reproductive isolation and morphological discontinuities in allopatry may not be fully synchronized (Wu 2001; De Queiroz 2007; Liu 2016), most units exhibiting morphological discontinuities may show varying degrees of reproductive isolation (Mallet 2008). In addition, geographical discontinuity is itself an important isolating mechanism that maintains morphological and genetic boundaries (Schluter 2001). In this study, Cluster B (*R. caudata*) is restricted to southern China (Guangdong), whereas Cluster C is distributed in South Korea, the Liuchiu (Ryukyu) Islands, and Japan (Fig. 5). Clearly, these two distinct clusters are geographically discontinuous, and no evidence suggests that the two widely recognized species should be merged into a single species. Therefore, the recognition of Cluster B (corresponding to *R. caudata*) and Cluster C (corresponding to *R. franguloides* and its synonym from Japan) as separate species should remain unchanged (Figs 1, 2, 3, 4, 5; Table 4). Furthermore, Cluster A differs considerably in morphology from Clusters B and C (Fig. 4; Table 4), with B and C hypothesized to be the most closely related pair, and Cluster A is also largely or completely allopatric with respect to both (Fig. 5). The Cluster A was previously described as *R. obovalis* or *B. congesta*. *Berchemia congesta* (1875) predates *R. obovalis* (1914) (Chen and Schirarend 2007). Under the rules of nomenclature, *Rhamnella congesta* should be the legitimate name for Cluster A, with *B. congesta* as the homotypic synonym and *R. obovalis* as its heterotypic synonym. Based on the morphological evidence and geographical distributions, these three major clusters identified here correspond well to *R. congesta*, *R. caudata*, and *R. franguloides*. The two varieties (var. *franguloides* and var. *inaequilatera*) of the newly-delimited *R. franguloides* can be distinguished only by leaf length (Table 4). Although they exhibit pronounced geographical separation (Fig. 5), there is no other new evidence to support the elevation of var. *inaequilatera* to species rank. Thus, the existing taxonomic treatment of the two varieties is retained.

Overall, our morphological delimitation of this complex differs markedly from previous taxonomic treatments in terms of taxon descriptions, circumscriptions and geographical distributions, highlighting the importance of population‐level analyses of morphological variation for delimiting species boundaries and informing taxonomic decisions.

### Molecular evidence further supports the morphological delimitation

Molecular phylogenetic analysis is one of the most important approaches for inferring evolutionary relationships and delimiting species (e.g., Hu et al. 2015; Lu et al. 2024). Morphological evidence indicated that *R. caudata* and the newly-delimited *R. franguloides* were likely the most closely related pair in this complex. However, it is also necessary to identify the key morphological characters that reflect phylogenetic relationships. Our sequence alignment revealed many variable sites that clearly distinguish the three species (Table 5). Either *R. congesta* (Cluster A) or *R. caudata* (Cluster B) possessed five fixed diagnostic nucleotide sites compared to the other two species. In addition, *R. franguloides* (Cluster C) possessed nine private sites that distinguished it from either Cluster A or Cluster B, whereas Cluster B had ten private sites when compared with either Cluster A or Cluster C (Table 5). These findings suggested strong genetic divergence among these three clusters or species. Therefore, *R. caudata* (Cluster B) is one well-delimited species different from *R. franguloides* (Cluster C), by integrating multiple lines of evidence from morphology, geographic distribution, and genetic divergence. Furthermore, the phylogenetic tree showed that *R. congesta* (Cluster A) forms an independent clade sister to the well-supported clade containing *R. caudata* (Cluster B) and *R. franguloides* (Cluster C) (Fig. 6). This finding suggested that the absence or presence of petiole and annual branchlet pubescence, petiole length, and leaf apex length are congruent with the inferred phylogenetic relationships (Figs 5, 6; Table 4). Under an integrative framework for species delimitation (Hu et al. 2015; Liu 2016; Lu et al. 2024), *R. congesta* (Cluster A) as a species was strongly supported by morphological and molecular evidence.

It should also be noted that leaf petiole length can well reflect the phylogenetic relationship (Table 4; Fig. 6). The variety R. franguloides var. inaequilatera was originally described as a species (Ohwi 1951), only with the longer leaf petiole (7.8–15.8 mm) than var. *franguloides* (3.8–7.6 mm) (as redefined in this study, Table 4). Although morphological difference among species in Rhamnaceae are often subtle (Chen and Schirarend 2007), whether this variety should be reinstated at species rank remains unclear. If both ITS sequences are assigned to either variety, it is likely that var. *franguloides* is more closely related to *R. caudata*. The acceptance of this hypothesis would imply that *R. inaequilatera* should be resurrected. However, the present study cannot determine whether the downloaded ITS sequences of *R. franguloides* should be assigned to both varieties or either variety (Table 5). To resolve this, more samples of *R. franguloides* should be collected for molecular phylogenetic and morphological analyses. Additionally, the present study relied solely on ITS marker, and sampling of Cluster C remains limited. Further studies incorporating additional molecular markers and expanded sampling could help further clarify relationships and genetic divergences within the *R. franguloides* complex.

### Key to the three species in the *Rhamnella franguloides* complex (Chinese names are given in parentheses where available)

The following key is based on diagnostic characters identified from all specimens examined in the present study. These three species are most reliably distinguished by combinations of these characters. For the two varieties within *R. franguloides*, single leaf petiole length can separate them completely. For taxa with more than two characters listed in the key, we strongly recommend using all of them for field identification.

**Table:** 

1	Leaf petioles and annual branchlets pubescent; short leaf petioles (1.0–3.8 mm); short leaf apices (2.2–9.0 mm)	***R. congesta* (**中华猫乳)
–	Leaf petioles and annual branchlets glabrous or nearly glabrous; long leaf petioles (3.5–15.8 mm); long leaf apices (7.8–24.0 mm)	**2**
2	Old branchlet (ca. 2 mm in diameter) lenticels dense (87–144 per cm^2^); large dry fruit (9.0–10.9 mm × 3.6–4.4 mm)	***R. caudata* (**尾叶猫乳)
–	Old branchlet (ca. 2 mm in diameter) lenticels sparse (54–87 per cm^2^); small dry fruit (5.5–8.7 mm × 2.9–4.0 mm)	**3 (*Rhamnella franguloides* )**
3	Leaf petioles 3.8–7.6 mm	**R. franguloides var. franguloides**
–	Leaf petioles 7.8–15.8 mm	**R. franguloides var. inaequilatera**

## Taxonomic treatment

### 1. Rhamnella congesta

Taxon classificationPlantaeRosalesRhamnaceae

(S.Moore) Z.Qiang Lu & X.L.Zhou comb. nov.

6753AECB-B227-5392-8EC8-CFDB667A91B4

urn:lsid:ipni.org:names:77396888-1

≡ Berchemia congesta S.Moore, J. Bot. 13: 226 (1875). Type: China.• Shanghai: 1860, A.C. Maingay 707 (syntype: K, barcode K000723049!). **Basionym**
= Rhamnella obovalis C.K.Schneid. in Sarg., Pl. Wilson. 2(1): 223 (1914), syn. nov.

#### Type.

**China. Hubei**: • Fang Hsien (Fangxian), July 1907, E.H. Wilson 3389 (holotype: A, barcode A00032772!; isotype: US, barcode US00094390!). ***Paratype*: China**. • **Jiangxi**: 27 July 1907, E.H. Wilson 1515 (E, barcode E00275330!).

#### Diagnosis.

It is mainly distinguishable from both *R. caudata* and *R. franguloides* by its pubescent leaf petioles and annual branchlets, shorter leaf petioles, 1.0–3.8 mm (mean 1.5–3.1 mm), and shorter leaf apices, 2.2–9.0 mm (Table 4).

#### Distribution.

Central, eastern, and southeastern China, rarely extending to South Korea (Fig. 5).

#### Specimens examined.

**China. Anhui**: • Shexian, 25 Aug 1958, Anonymous 60 (PE 00093012); • Huoshan, near Maoshan, 30 Jul 1953, East-China Work Station 6637 (KUN 0417957). Henan: • Tongbai County, 11 Oct 2012, Jinting Dong HNP059 (AU AU078090); • Funiu Shan, Sep 1958, Yuanrong Pei 121 (HENU 0350389); • Jigong Shan, 1 Jul 1990, Forestry Class 88 903075 (CCAU ccau0004631); • Pingqiao District, Xinyang, 19 Jul 2021, Yi Cheng DC563 (HIB HIB0220908). **Jiangsu**: • Baoyan Shan, Yushan, 19 Aug 1958, Wenxiang Wu 4596 (WUK 0209822); • Sucheng District, Lianyungang, s.d., Zenglai Xu 842 (NAS NAS00620242). **Jiangxi**: • Taohongling National Nature Reserve, Pengze County, 17 Jul 2015, Enxiang Li et al. 15070758 (JJF JJF00022517). **Shaanxi**: • Ankang, 25 Aug 1959, Peiyuan Li 11039 (WUK 0140791). **Shandong**: • Guanshui Town, Mouping District, Yantai, 16 Sep 2012, Zhongli Wang 604010026 (SDFGR SDF1007616). **Shanghai**: • s.d., Anonymous s.n. (PE 00093047). **Zhejiang**: • Longjing, 23 Aug 1955, Xianyu He 1249 (HHBG HZ025991).

#### Note.

This species corresponds to Cluster A, as redefined in this study, and the other examined specimens of this species can be found in the Suppl. material 1 Because *Berchemia congesta* (published in 1875) predates *R. obovalis* (published in 1914), the epithet *congesta* has priority, and the new combination is proposed here.

### 2. Rhamnella caudata

Taxon classificationPlantaeRosalesRhamnaceae

Merr., Sunyatsenia 2: 11 (1934)

81F72F73-8013-5151-9AC7-6BBFFE7B94DF

#### Type.

**China. Guangdong**: • Yuyuen (Ruyuan), 28 May 1933, S.P. Ko 52749 (holotype: NY, barcode NY00415048!; isotypes: A, barcode A00051373!; PE, barcode PE00023560!). ***Paratype*: China. Guangdong**: • Ruyuan, 17 July 1933, S.P. Ko 53085 (PE, barcode PE00023559!).

#### Diagnosis.

This species resembles *R. franguloides* in having glabrous or nearly glabrous petioles and annual branchlets, but differs from it in having denser lenticels (87–144 per cm^2^ on annual branchlets ca. 2 mm in diameter) and larger dry fruits (9.0–10.9 × 3.6–4.4 mm).

#### Distribution.

Ruyuan, Guangdong, China (Fig. 5).

#### Specimens examined.

**China. Guangdong**: • Ruyuan, 28 May 1933, S.P. Ko 52749 (IBK IBK00174838; SN SN006379); • Ruyuan, 17 Jul 1933, S.P. Ko 53085 (NAS NAS00071549; IBK IBK00174837); • Ruyuan, 2025, X.L. Zhou 2025RY01–2025RY13 (XJU).

#### Notes.

This species corresponds to Cluster B in this study; its morphological boundaries are redefined here (Table 4). No synonyms are currently recorded for this name (IPNI 2026; POWO 2026).

### 3. Rhamnella franguloides

Taxon classificationPlantaeRosalesRhamnaceae

(Maxim.) Weberb., Nat. Pflanzenfam. 3(5): 406 (1895)

B7FA9E7E-BAE4-57EF-AD9B-47D204835199

≡ Microrhamnus franguloides Maxim., Mém. Acad. Imp. Sci. St.-Pétersbourg, Sér. 7, 10(11): 4, t. 1, f. 13–15 (1866). Type: Japan. • Kyushu: Prov. Higo, 1863, C.J. Maximowicz s.n. (syntypes: K, barcode K000723046!; S, barcode S-G-5219!).
= Rhamnella japonica Miq., Ann. Mus. Bot. Lugduno-Batavi 3: 30 (1867).

#### Type.

**Japan. Nagasaki**: • 1862, R. Oldham 165 (lectotype: L, barcode L0553944!, designated by M.C. Johnston in sched., 1969).

#### Diagnosis.

This species differs from *R. congesta* in having glabrous or nearly glabrous leaf petioles and annual branchlets, longer leaf apices (7.8–24.0 mm), and longer leaf petioles 3.8–15.8 mm (mean 4.4–13.6 mm), and from *R. caudata* in having sparser lenticels (54–87 per cm^2^ on annual branchlets with ca. 2 mm in diameter), and smaller dry fruits (5.5–8.7 × 2.9–4.0 mm).

#### Distribution.

Korea, the Liuchiu (Ryukyu) Islands and Japan (Fig. 5).

#### Note.

This species corresponds to Cluster C in this study and comprises two distinguishable varieties. The type specimen of *Microrhamnus taquetii* (barcode No. A00003587; voucher: *T. Taquet 153*), the annotation by Kim (February 1993) on the type sheet identified as *E. alatus*, had been treated as a synonym of *E. alatus* by Kim (1993) and Ma (2001). Subsequently, it was treated as a synonym of *R. franguloides* (Chen and Schirarend 2007). Our examination of the type specimen of *M. taquetii* (A00003587 in Fig. 1A) shows that it has opposite leaves and branches, branches with at least two longitudinal corky wings, subquadrangular branchlets, and rounded winter buds with irregularly and sharply denticulate scale margins. These morphological characteristics are diagnostic of *E. alatus* (Celastraceae), which is consistent with the treatment of Kim (1993) and Ma (2001). Therefore, *M. taquetii* should be excluded from the synonymy of *R. franguloides*. We support the treatment of Kim (1993) and Ma (2001), and the annotation by Kim (February 1993) on the type sheet.

### 3a. Rhamnella franguloides var. franguloides

Taxon classificationPlantaeRosalesRhamnaceae

A0142C48-86AC-5278-9D8F-F78CD8036E38

#### Distribution.

Korea and Japan (Fig. 5).

#### Specimens examined.

**Japan. Honshu**: • Okayama Pref. (Prov. Bitchu), Atetsu-gun, Izumi, 30 Jul 1962, *G. Murata* 27259 (US US2409922). • Yamaguchi, Abugun, Chomonkyo, 20 Jun 1927, Saito S s.n. (L L.4197492). Kyushu: • Nagasaki, 1862, R. Oldham 165 (M M-0211847); • Kiusiu, Prov. Higo, 1863, C. J. Maximowicz s.n. (L L.2324928; US US16608); • Miyazaki Pref., Miyazaki city, Ukinojo, 12 Jun 1926, N. Eri 100568 (US US1347299); • Miyazaki-ken, Mt. Taki-san, 31 Jul 1946, I. Hurusawa 205 (US US2073776). **Korea. Gwangju**: • Mt. Mudeung, 11 Sep 2012, *S.-Y. Jung & H.-S. Hwang* P126947 (PE 02090069). **Jeollabuk-do**: • Buan-gun, Byeonsan Peninsula, 7 Aug 1992, *D.E. Boufford, T.J. Kim, C.W. Park & B.Y. Sun* 25800 (KUN 0452551).

#### Note.

The other examined specimens of this variety can be found in the Suppl. material 1, and all examined specimens of var. *inaequilatera* are listed in its treatment.

### 3b. Rhamnella franguloides var. inaequilatera

Taxon classificationPlantaeRosalesRhamnaceae

(Ohwi) Hatus., Sci. Bull. Agric. Div. Univ. Liuchiu (Ryukyu) 3: 21 (1956)

9F6DC862-1CB6-5F61-BCD5-C4169C71F21E

≡ Rhamnella inaequilatera Ohwi, J. Jap. Bot. 26(8): 231 (1951).

#### Type.

**Insula Ishigaki, the Liuchiu (Ryukyu) Islands**: July 2, 1891, *S. Tanaka* 274 (holotype: TNS, catalogue number 60478).

#### Diagnosis.

The two varieties of *R. franguloides* are distinguished by petiole length (3.8–7.6 mm in var. *franguloides* vs. 7.8–15.8 mm in var. *inaequilatera*).

#### Distribution.

The Ryukyu Islands (Fig. 5).

#### Specimens examined.

**The Liuchiu (Ryukyu) Islands**: • Kagoshima Pref., Aira-gun, Yoshimatsu-cho, 7 Sep 2001, *S. Fujii* 8789 (L L.4207352); • Miyazaki Pref., Shintomi-machi, 12 Sep 1980, *J. Murata* 10020 (WAG WAG.1250662; L L.2324930); • Tokunoshima, interior of Boma, Tokunoshima-cho, Oshima-gun, Kagoshima Pref., 28 Aug 1975, K. Iwatsuki et al. 450 (L L.2324938); • Tokunoshima, interior of Shikaura, Isen-chō, Ōshima-gun, Kagoshima Pref., 26 Aug 1975, K. Iwatsuki et al. 426 (L L.2324939). • Okinawa-honto, Shoshi, Nakijin-mura, Kunigami, 14 May 1955, S. Hatusima 17814 (L L.2324941); • Okinawa-honto, Motobu-cho, Tancha, 21 Jun 1966, *S. Tawada* s.n. (L L.2324931); • Okinawa-honto, Kunigami-son, Mt. Hedoishi, 12 Apr 1974, *M. Furuse* 5580 (K K004542034); • Okinawa-honto, Kunigami-son, Yona, 11 Apr 1974, *M. Furuse* 5553 (K K004542033); • same locality, 4 Nov 1972, *M. Furuse* 1742 (K K004542032); • Okinawa-honto, Nago City, Haneji, Makiya, 20 Aug 1980, Y. Miyagi 9151 (BISH BISH1255065). • Amami-oshima, Ohagachi, Tatugomura, 14 Aug 1956, S. Hatusima 20310 (L L.2324940); • Amami-oshima, Kasari-cho, Mt. Yodo, 7 Aug 2020, Anonymous s.n. (KAG KAG024857); • Amami-oshima, Yamato-mura, 20 Jun 2017, Anonymous s.n. (KAG KAG019217); • Amami-oshima, Tatsugo-cho, Nagakumo Pass, 4 Aug 2020, Anonymous s.n. (KAG KAG023984).

#### Note.

The holotype of var. *inaequilatera* was collected by *S. Tawada* (collection information: *S. Tawada*) from Insula Ishigaki (24.5°N, 124.3°E). We could not find this specimen for direct morphological measurement. Therefore, the distribution range of this variety is significantly larger than that shown in Fig. 5. However, strong geographic isolation still exists between two variety and between var. *inaequilatera* and any other one species in this complex.

## Supplementary Material

XML Treatment for Rhamnella congesta

XML Treatment for Rhamnella caudata

XML Treatment for Rhamnella franguloides

XML Treatment for Rhamnella franguloides var. franguloides

XML Treatment for Rhamnella franguloides var. inaequilatera

## Additional information

### Artificial Intelligence (AI) use

The authors accept full responsibility for the content of the manuscript, including the disclosure of any use of AI.

The English of this manuscript was polished with the assistance of DeepSeek; the authors take full responsibility for the content of the publication.

### Author ORCIDs

Q. An https://orcid.org/0009-0005-1745-6877

Z. Lu https://orcid.org/0000-0002-6169-4849

X. Zhou https://orcid.org/0009-0009-4372-247X

### Data availability

All of the data that support the findings of this study are available in the main text or Supplementary Information.

## Supplementary materials

Supplementary material 1 Specimen information, sampling localities, type specimens, sequence information and a morphological trait diagram for the *Rhamnella franguloides* complex Data type docx Explanation note **table SS1**. Specimen information and database sources of the herbarium specimens in Fig. 1. **table SS2**. Sampling information (collector, collection number, date, locality, barcode, and herbarium) of the specimens used for PCA in *Rhamnella franguloides* complex. Note: The Coordinate source column indicates how the mapping coordinates (Fig. 2) were obtained from each specimen (‘label’ = coordinates printed on the label; ‘georeferenced’ = coordinates inferred from the locality on the label). Coordinates of the newly collected populations in Table 1 were recorded by GPS. **table SS3**. Type specimens examined for PCA and morphological comparison in this study. **table SS4**. Individual-level sequence information for all newly collected samples. **fig. S1**. Diagram of leaf partial morphological traits in Tables 2, 3. https://binary.pensoft.net/file/1779795This dataset is made available under the Open Database License (http://opendatacommons.org/licenses/odbl/1.0/). The Open Database License (ODbL) is a license agreement intended to allow users to freely share, modify, and use this Dataset while maintaining this same freedom for others, provided that the original source and author(s) are credited. Qianna An, Zhiqiang Lu, Xiaolong Zhou
