# Supplementary material for: Species delimitation and taxonomic revision of the Rhamnella franguloides complex (Rhamnaceae)
Source: PhytoKeys. 2026 Sep 18;279:179–200. doi: 10.3897/phytokeys.279.204054 (PMC13615397; doi:10.3897/phytokeys.279.204054)
Supplement: Supplementary file 1 [file phytokeys-279-179_article-204054__-s001.docx]

**Species delimitation and taxonomic revision of the *Rhamnella franguloides* complex (Rhamnaceae)**

Qianna An^1^, Zhiqiang Lu^2*^, Xiaolong Zhou^1*^

^1. College of Ecology and Environment, Xinjiang University, Urumqi 830046, China^

^2. Xishuangbanna Tropical Botanical Garden of Chinese Academy of Sciences, Mengla 666303, China^

^*Corresponding author: Xiaolong Zhou (E-mail:^ [^zhouxiaolong@xju.edu.cn^](mailto:zhouxiaolong@xju.edu.cn)^), Zhiqiang Lu (^[^luzhiqiang@xtbg.ac.cn^](mailto:luzhiqiang@xtbg.ac.cn)^)^

**Supplementary tables and figure**

**Table S1.** Specimen information and database sources of the herbarium specimens in Figure 1.

| **Figure label** | **Specimen information** | **Source & Citation** |
| --- | --- | --- |
| A | Type specimen of *Microrhamnus* *taquetii* (barcode: A00003587) | Kennedy J (2026). Harvard University Herbaria: All Records. Harvard University Herbaria. Occurrence dataset https://doi.org/10.15468/o3pvnh accessed via GBIF.org on 2026-06-15.  <https://www.gbif.org/occurrence/1999008802> |
| B | Representative specimen of *Euonymus* *alatus*  (barcode: PE01700680) | Chinese Virtual Herbarium (CVH). (https://www.cvh.ac.cn). |
| C | Type specimen of *Euonymus* *alatus* var. *apertus* Loes. (barcode: A00003586) | Kennedy J (2026). Harvard University Herbaria: All Records. Harvard University Herbaria. Occurrence dataset https://doi.org/10.15468/o3pvnh accessed via GBIF.org on 2026-06-15.  <https://www.gbif.org/occurrence/1998730426> |
| D | Syntype specimen of *Rhamnella* *franguloides* var. *franguloides* (barcode: K000723046) | Royal Botanic Gardens, Kew (2026). Royal Botanic Gardens, Kew - Herbarium Specimens. Occurrence dataset https://doi.org/10.15468/ly60bx accessed via GBIF.org on 2026-06-15.  <https://www.gbif.org/occurrence/912560758> |
| E | Holotype specimen of *Rhamnella* *obovalis*  (barcode: A00032772) | Kennedy J (2026). Harvard University Herbaria: All Records. Harvard University Herbaria. Occurrence dataset https://doi.org/10.15468/o3pvnh accessed via GBIF.org on 2026-06-15.  <https://www.gbif.org/occurrence/1999269253> |
| F | Syntype specimen of *Berchemia* *congesta* S.Moore.  (barcode: K000723049) | Royal Botanic Gardens, Kew (2026). Royal Botanic Gardens, Kew - Herbarium Specimens. Occurrence dataset https://doi.org/10.15468/ly60bx accessed via GBIF.org on 2026-06-15.  https://www.gbif.org/occurrence/912560752 |
| G | Lectotype specimen of *Rhamnella* *japonica* Miq.  (barcode: L0553944) | Bijmoer R, Arias Guerrero S, Creuwels J (2026). Naturalis Biodiversity Center (NL) - Botany. Naturalis Biodiversity Center. Occurrence dataset https://doi.org/10.15468/ib5ypt accessed via GBIF.org on 2026-06-15.  https://www.gbif.org/occurrence/2513241674 |
|  |  |  |
| H | Paratype of *Rhamnella* *caudata* Merr. & Chun.  (barcode: PE00023559.) | Chinese Virtual Herbarium (CVH).  (https://www.cvh.ac.cn). |

**Supplementary Table S2.** Sampling information (collector, collection number, date, locality, barcode, and herbarium) of the specimens used for PCA in *Rhamnella franguloides* complex

| **Collector** | **Collection No.** | **Date** | **Locality** | **Barcode** | **Herbarium** | **Coordinate source** |
| --- | --- | --- | --- | --- | --- | --- |
| 86 Forestry | 59 | 1988-07-01 | Hubei, China | ccau0004636 | CCAU | georeferenced |
| 86 Forestry | 86-02-006 | 1988-10-01 | Hubei, China | ccau0004641 | CCAU | georeferenced |
| 86 Forestry | 8602006 | 1988-10-01 | Hubei, China | ccau0004637 | CCAU | georeferenced |
| 88 Forestry | 87 | 1990-07 | Henan, China | ccau0004610 | CCAU | georeferenced |
| 88 Forestry | 901-131 | 1990-07-01 | Henan, China | ccau0004608 | CCAU | georeferenced |
| 88 Forestry | 903075 | 1990-07-01 | Henan, China | ccau0004631 | CCAU | georeferenced |
| 88 Forestry | 905159 | 1990-07-01 | Henan, China | ccau0004614 | CCAU | georeferenced |
| A. N. Steward | 9769 | 1925-08-03 | Henan | US 1370470 | US | georeferenced |
| An Zuojun | T00088 | 1985-07-16 | Henan, China | 01637964 | PE | georeferenced |
| An Zuojun | T00088 | 1985-07-16 | Henan, China | 01637965 | PE | georeferenced |
| Anonymous | 016 | 1957.08. | Henan, China | 350375 | HENU | georeferenced |
| Anonymous | 0530 | 1986-07-20 | Anhui, China | 01636355 | PE | georeferenced |
| Anonymous | 0762 | 1958-08-07 | Zhejiang, China | 93024 | PE | georeferenced |
| Anonymous | 1025 | — | Shanghai, China | 00093046 | PE | georeferenced |
| Anonymous | 1159 | 1958-07-24 | Zhejiang, China | HZ026035 | HHBG | georeferenced |
| Anonymous | 1281 | 1957-08-22 | Zhejiang, China | HZ025976 | HHBG | georeferenced |
| Anonymous | 15740 | 1958-05-23 | Jiangsu, China | HZ026026 | HHBG | georeferenced |
| Anonymous | 20436 | 1958-08-20 | Jiangsu, China | HZ026012 | HHBG | georeferenced |
| Anonymous | 2698 | 1958-06-13 | Zhejiang, China | HZ025978 | HHBG | georeferenced |
| Anonymous | 27404 | 1957-08-07 | Zhejiang, China | HZ026038 | HHBG | georeferenced |
| Anonymous | 27404 | — | Zhejiang, China | HZ026039 | HHBG | georeferenced |
| Anonymous | 3366 | 1959-08-01 | Anhui, China | 93013 | PE | georeferenced |
| Anonymous | 390 | 1959-07-06 | Anhui, China | NAS00578108 | NAS | georeferenced |
| Anonymous | 390 | 1960-08-23 | Anhui, China | NAS00578109 | NAS | georeferenced |
| Anonymous | 53085 | 1933-07-17 | Guangdong, China | IBK00174837 | IBK | georeferenced |
| Anonymous | 537 | 1958-06-16 | Zhejiang, China | HZ025986 | HHBG | georeferenced |
| Anonymous | 60 | 1958-08-25 | Anhui, China | 93012 | PE | georeferenced |
| Anonymous | 6548 | 1986-06-22 | Anhui, China | 01637374; 1637374 | PE | georeferenced |
| Anonymous | 702 | 1958-07-06 | Zhejiang, China | HZ026037 | HHBG | georeferenced |
| Anonymous | 762 | 1958-08-07 | Zhejiang, China | HZ025992 | HHBG | georeferenced |
| Anonymous | 7833024 | 1979-09-01 | Zhejiang, China | SWFC0006656 | SWFC | georeferenced |
| Anonymous | 79 | 1983-08-04 | Henan, China | 01637959 | PE | georeferenced |
| Anonymous | 877045 | 1987-07-01 | Henan, China | PEY0036594 | PEY | georeferenced |
| Anonymous | 9 | 1960-08-11 | Anhui, China | NAS00578064 | NAS | georeferenced |
| Anonymous | D508 | 1922-09-17 | Zhejiang, China | 93038 | PE | georeferenced |
| Anonymous | Fan0042 | 1982-05-24 | Zhejiang, China, Huoyanshan | ZMNH0046873 | ZM | georeferenced |
| Anonymous | II0055 | 1958-07-25 | Shaanxi, China | 1637258 | PE | georeferenced |
| Anonymous | — | 1930-07-22 | Zhejiang, China | PEY0036597 | PEY | georeferenced |
| Anonymous | — | — | Jiangsu, China | 92826 | PE | georeferenced |
| Anonymous | — | — | Shanghai, China | 93047 | PE | georeferenced |
| Beijing Normal University Class of 1998 | 1-125 | 2000-07-21- | Shandong, China | 0020385; 20385 | BNU | georeferenced |
| Bi Yuke et al. | BYK1701 | 2012-06-27 | Zhejiang, China | CSH0057338 | CSH | label |
| C. S. Fan & Y. Y. Li | 120 | 1934-08-05 | Anhui, China | US 1756442 | US | georeferenced |
| C. Y. Chiao | 18704 | 1928-07-25 | Jiangxi, China | US 1553978 | US | georeferenced |
| C. Y. Chiao | 2637 | 1930-06-29 | China | US 1553789; US 1575904 | US | georeferenced |
| C.T.Ren | — | 1926-08-26 | Jiangsu, China | PEY0036600 | PEY | georeferenced |
| Cai Jie,Zhang Ting,Dong Zhenguo | 12CS5439 | 2012-07-24 | Jiangsu, China | 1330260 | PE | label |
| Cai Ruyi, Xi Ya, Zhang Liping | 14257 | 2014-07-19 | Jiangxi, China | JJF00022514 | JJF | georeferenced |
| Cai Xiuzhen, Li Jun, Kuang Renping, Chen Lingling, Cao Zhouhong | CXZ556 | 2021-08-01 | Henan, China | HIB0215327 | HIB | label |
| Cai Xiuzhen, Li Jun, Kuang Renping, Chen Lingling, Cao Zhouhong | CXZ603 | 2021-08-02 | Henan, China | HIB0223207 | HIB | label |
| Cai Xiuzhen, Li Jun, Wang Ting | CXZ62 | 2020-08-18 | Henan, China | HIB0206288 | HIB | label |
| Census Specimen | 14354 | 1905-05-12 | Henan, China | 0417959 | KUN | georeferenced |
| Census Specimen | 14534 | 1959 | Henan, China | 01637383 | PE | georeferenced |
| Chang-Soo Kim & Sang-Hun Oh | 1560 | 1995-08-24 | Korea, Jeonnam-do, Yeongam-gun, Wolchulsan | GA 190649 | GA | georeferenced |
| Chen Yansong, Chen Cuibing, Shen Yun | XuZD053 | 2009-07-11 | Anhui, China | 1330263 | KUN | label |
| Chen Yilin | 84-13 | 1984-08-12 | Jiangxi, China | 01878756 | PE | georeferenced |
| Chen Yilin | 84-14 | 1984-08-19 | Jiangxi, China | 01878753; 01878754 | PE | georeferenced |
| Chen Zhiyuan, Wang Changqing | 006 | 1982-09-24 | Hubei, China | ccau0004612 | CCAU | georeferenced |
| Chen Zhiyuan, Wang Changqing | 6 | 1982-09-24 | Hubei, China | ccau0004611; ccau0004613; ccau0004616 | CCAU | georeferenced |
| Cheng Yi | DC563 | 2021-07-19 | Henan, China | HIB0220908 | HIB | label |
| Chiao, C.Y. | 2637 | 1930-06-29 | China | K004542052; K004542053 | K | georeferenced |
| Ching, R.C. | 2928 | 1925-07-05 | Anhui, China | K004542051 | K | georeferenced |
| Collector unknown | s.n. | 1910-08-14 | Japan | US 1156502 | US | georeferenced |
| D. E. Boufford | 25800 | 1992-08-07 |  | 73746 | HAST | georeferenced |
| D.E.Boufford et al. | 25800 | 1992-08-07 | Korea, Chollabuk-do, Puan-gun, Sangseo-Myun | KUN0097897/HUH | KUN/HUH | georeferenced |
| D.E.Boufford, T.J.Kim, C.W.Park, B.Y.Sun | 25800 | 1992-08-07 | Korea, Chollabuk-do, Puan-gun, Sangseo-Myun | KUN0097897; PE1695254 | KUN/PE/HUH | georeferenced |
| D.E.Boufford, T.J.Kim,C.W.Park&B.Y.Sun | 25800 | 1992-08-07 | Korea | 0452551 | KUN | georeferenced |
| Deng Maobin, Liu Fangxun, Huang Zhiyuan | 2607 | 1956-09-11 | Jiangsu, China | 196854 | WUK | georeferenced |
| Dong Anmiao | TCM289 | 2010-09-18 | Jiangxi, China | JJF00022512 | JJF | georeferenced |
| Dong Jinting | HNP059 | 2012-10-11 | Henan, China | AU078090 | AU | georeferenced |
| E. H. Walker | 8402 | — | Japan | US 2619475 | US | georeferenced |
| E. H. Wilson | 3389 | 1907-07-01 | Hubei, China | US 599880; barcode-00032772 | A/US | georeferenced |
| E. Hatusima | 20110 | 1934-08-24 | Japan, Amamioshima, Ohmoricho, Fukyama | L.204940 | L | georeferenced |
| E. Hatusima | 79 | 1951-09-07 | Japan, Ryukyu, Okinawa, Kunigami-gun | K004542017; US 2093103 | K/US | georeferenced |
| E. Wilson | 1515 | 1907-07-27 | Jiangxi, China | US 599325 | US | georeferenced |
| E. Wilson | 9365 | 1917-10-28 | Korea, Republic of | US 1052034 | US | georeferenced |
| E.J. Taquet | Taquet 3214 | 1909-08 | Korea, Republic of | FKP:89624 | A | georeferenced |
| E.J. Taquet | Taquet 640 | 1908-08-22 | Korea, Republic of | FKP:88117 | A | georeferenced |
| East China Ministry of Agriculture and Forestry Forest Survey Zhejiang Team | 40005 | 1950-07-22 | Zhejiang, China | CAF00024556 | CAF | georeferenced |
| East China Workstation Staff | 6637 | 1953-07-30 | Anhui, China | 417957 | KUN | georeferenced |
| East China Workstation | 6637 | 1953-07-30 | Anhui, China | 93011 | PE | georeferenced |
| Economics Class of 1980 | J8012184 | 1982-09-22 | Zhejiang, China | CSFI023795 | CSFI | georeferenced |
| Egbert H. Walker | 8402 | 1966-06-24 | Japan, Okinawa, Gogaiyama | L.0553943 | L | georeferenced |
| Fan CS; Li YY | Fan, CS; Li, YY 125 | 1936-06-17 | China | L.2324944 | Botany | georeferenced |
| Fan, C.S.; Li, Y.Y. | 120 | 1934-08-05 |  | K004542054 | K | georeferenced |
| Fang Wenzhe et al. | 103 | 1962-06-29 | Jiangsu, China | 220444 | WUK | georeferenced |
| Fang Wenzhe et al. | 103 | 1962-05-28 | Jiangsu, China | IBK00174839 | IBK | georeferenced |
| Fang Wenzhe et al. | 193 | 1963-06-21 | Anhui, China | 220841; IBK00174842 | IBK/WUK | georeferenced |
| Fang Wenzhe et al. | 97 | 1960-08-14 | Jiangsu, China | 219273; IBK00325947 | IBK/WUK | georeferenced |
| Forbes FB | Forbes, FB s.n. | 1879-06-01 | China | L.2324942 | Botany | georeferenced |
| Forbes FB | Forbes, FB s.n. | 1881-06-05 | China | L.2324943 | Botany | georeferenced |
| Forestry Class of 1981 | 83092 | 1983-06-04 | Henan, China | 00121757; 00121758 | BJFC | georeferenced |
| Fujii S | Fujii, S 8789 | 2001-09-07 |  | L.4207352 | Botany | georeferenced |
| Funiu Mountain Survey Team | 150 | 1961-08-31 | Henan, China | 01470859 | PE | georeferenced |
| Furuse, M. | 1742 | 1972-11-04 |  | K004542032 | K | georeferenced |
| Furuse, M. | 43123 | 1965-06-07 | Japan | K004542056 | K | georeferenced |
| Furuse, M. | 5553 | 1974-04-11 |  | K004542033 | K | georeferenced |
| Furuse, M. | 5580 | 1974-04-12 |  | K004542034 | K | georeferenced |
| G. Koidzumi | — | 1905-07-30 | Japan, Honshu, Bitchu-Ikuni, Soushiu | US 2109922 | US | georeferenced |
| G. Murata | 27259 | 1962-07-30 | Japan | US 2409922 | US | georeferenced |
| Gao Shuangju | 672 | 2012-08-27 | Shandong, China | SDF1007628 | SDFGR | georeferenced |
| Gao Xipeng | 52749 | 1933-05-28 | Guangdong, China | 00023560; IBK00174838 | IBK/PE | georeferenced |
| Gao Xipeng | 52749 | 1933-05-28 | Guangdong, China | SN006379 | SN | georeferenced |
| Gao Xipeng | 53085 | 1933-07-17 | Guangdong, China | 00023559; NAS00071549 | NAS/PE | georeferenced |
| Geng Jiazhen | 85 | 2012-07-17 | Shandong, China | SDF1007624 | SDFGR | georeferenced |
| Group 3 | 3059 | 1989-08-01 | Hubei, China | ccau0004625 | CCAU | georeferenced |
| Group 3 | 3059 | 1989-08 | Hubei, China | 8221 | CCAU | georeferenced |
| Group 4 | 4042 | 1989-08-01 | Hubei, China | ccau0004624; ccau0004640 | CCAU | georeferenced |
| Group 5 | 5-003 | 1989-08-01 | Hubei, China | ccau0004642 | CCAU | georeferenced |
| Group 5 | 5-033 | 1989-08-01 | Hubei, China | ccau0004628 | CCAU | georeferenced |
| Group 5 | 55-033 | 1989-08 | Hubei, China | ccau0004618 | CCAU | georeferenced |
| Guo Benzhao | 3692 | 1958-09-16 | Shaanxi, China | 0113017 | WUK | georeferenced |
| Guo Benzhao | 3717 | 1958-09-26 | Shaanxi, China | 395108 | WUK | georeferenced |
| Guo Wenxiang | 3739 | 1958-07-25 | Zhejiang, China | 01637378 | PE | georeferenced |
| Han Xiaoqing | 5112039 | 2012-08-17 | Shandong, China | SDF1007614 | SDFGR | georeferenced |
| Hangzhou Botany | 1217 | 1958-09-26 | Zhejiang, China | 0177558 | WUK | georeferenced |
| Hatusima S | Hatusima, S 17814 | 1955-05-14 | Japan | L.2324941 | Botany | georeferenced |
| Hatusima S | Hatusima, S 20310 | 1956-08-14 | Japan | L.2324940 | Botany | georeferenced |
| He Shanbao | 343 | 1958-09-21 | Shaanxi, China | 101965 | WUK | georeferenced |
| He Xianyu | 1203 | 1953-04-01 | Zhejiang, China | HZ025990 | HHBG | georeferenced |
| He Xianyu | 1249 | 1955-08-23 | Zhejiang, China | HZ025991 | HHBG | georeferenced |
| He Xianyu | 1249 | 1955-08/ | Zhejiang, China | 0168610; 0181240 | WUK | georeferenced |
| He Xianyu | 22370 | 1957-06-27 | Zhejiang, China | HZ026002 | HHBG | georeferenced |
| He Xianyu | 23923 | — | Zhejiang, China | HZ026001 | HHBG | georeferenced |
| He Xianyu | 23979 | 1957-07-20 | Zhejiang, China | HZ026000 | HHBG | georeferenced |
| He Xianyu | 24723 | 1957-07-03 | Zhejiang, China | HZ025995 | HHBG | georeferenced |
| He Xianyu | 25270 | 1957-08-10 | Zhejiang, China | HZ025994 | HHBG | georeferenced |
| He Xianyu | 26212 | 1957-08-04 | Zhejiang, China | 0168685 | WUK | georeferenced |
| He Xianyu | 27406 | 1957-08-07 | Zhejiang, China | 0177686 | WUK | georeferenced |
| He Xianyu | 28243 | — | Zhejiang, China | HZ026034 | HHBG | georeferenced |
| Henan Agricultural College | 2413 | — | Henan, China | 0288803 | WUK | georeferenced |
| Henan Agricultural College | 2870 | — | Henan, China | 0288804 | WUK | georeferenced |
| Hers J. | 408 | 1919-05-22 | China | BR0000031816489 | BR | georeferenced |
| Hong Xin | ZhouSB0204 | 2012-08-11 | Anhui, China | 1330258 | KUN | label |
| Hou Yuantong, Guo Chengyong, Hou Chunli, Duan Gong | 12010870 | 2012-10-13 | Shandong, China | 3010 | QFNU | georeferenced |
| Hou Yuantong, Guo Chengyong, Hou Yuanmian, Sun Weiwei, Lv Xiaochen | 20170529459-6 | 2017-05-29 | Shandong, China | QFNU0037848 | QFNU | georeferenced |
| Hou Yuantong, Guo Chengyong, Wang Jing | 201408063-1 | 2014-08-29 | Hubei, China | QFNU0041472 | QFNU | label |
| Hou Yuantong, Guo Chengyong, Wang Jing | 201408063-2 | 2014-08-29 | Hubei, China | QFNU0041473 | QFNU | label |
| Hou Yuantong, Guo Chengyong, Wang Jing | 201408063-3 | 2014-08-29 | Hubei, China | QFNU0041474 | QFNU | label |
| Hou Yuantong, Guo Chengyong, Wang Jing | 201408063-4 | 2014-08-29 | Hubei, China | QFNU0041475 | QFNU | label |
| Hou Yuantong, Guo Chengyong, Wang Jing | 201408063-5 | 2014-08-29 | Hubei, China | QFNU0041476 | QFNU | label |
| Hou Yuantong | 11941 | 2011-07-28 | Shandong, China | QFNU0009358 | QFNU | label |
| Huang Yi | 6217 | 1958-07-20 | Jiangsu, China | HZ026025 | HHBG | label |
| Huangtu Team | 2542 | 1984-06-10 | Henan, China | 0456749 | WUK | georeferenced |
| Huangtu Team | 2775 | 1984-06-17 | Henan, China | 0456008 | WUK | georeferenced |
| Hwong & Su | — | 1930-10 | Japan, Tokyo | 00093043 | PE | georeferenced |
| I. Hurusawa | 205 | 1946-07-31 | Japan | US 2073776 | US | georeferenced |
| Institute of Botany 2016 Graduate Students | 048 | 2017-08-30 | Jiangsu, China | NAS00620272 | NAS | georeferenced |
| J. Murata | 10020 | 1980-09-12 | Japan, Miyazaki, Shintomi-machi, Minooshiro | L.204903/TI; PE1286664/TI; WAG.150960/TI | L/TI/PE/TI/WAG/TI | georeferenced |
| J.Murata | 10020 | 1980-09-12 | Japan | 0417979 | KUN | georeferenced |
| J.Murata | 10020 | 1980-09-12 | Japan | 00093045 | PE | georeferenced |
| JKS with C.S.Chang,G.I.Cheon | 1373 | 2003-09-27 | Korea | 1630199 | PE | georeferenced |
| JKS with C.S.Chang,G.I.Cheon | 1373 | 2003-09-27 | Korea | 1630199 | PE | georeferenced |
| Jiang Mingxi et al. | 2020081604 | 2020-08-16 | Hubei, China | HIB0208520 | HIB | georeferenced |
| Jiangpu Squadron | 8168 | 1958-07-02 | Jiangsu, China | 0209160 | WUK | georeferenced |
| Jiangsu Wild Plant Resource Survey Team, Nanjing Forestry Jurong Survey Team | 6217 | 1958-07-20 | Jiangsu, China | 210532 | WUK | georeferenced |
| Jiangsu Wild Plant Resource Survey Team, Nanjing Forestry Jurong Survey Team | 95 | 1958-07-28 | Jiangsu, China | 210389 | WUK | georeferenced |
| Jung Su-Young, Hwang Hee-Suk | — | 2012-09-11 | Korea, Mt.Mudeungsan, Gwangju | PE2504324/KH | PE/KH | georeferenced |
| Jung Su-Young,Hwang Hee-Suk | P126947 | 2012-09-11 | Korea | 02090069 | PE | georeferenced |
| K.M.Liou | 4372 | 1935-06-18 | Henan, China | 9404 | WUK | georeferenced |
| K.M.Liou | 8981 | 1938-08-04 | Hubei, China | 54576 | WUK | georeferenced |
| Kazunobu Ochi & Misao Natsui | 17 | 2015-06-13 | Japan, Okayama, Kasaiyama | PE02473394/TNS | PE/TNS | georeferenced |
| Lai Shushen et al. | 4233 | 1998-06-27 | Jiangxi, China | NAS00620328 | NAS | georeferenced |
| Lai Shushen et al. | 6826 | 1999-06-07 | Jiangxi, China | NAS00615395; NAS00615396 | NAS | georeferenced |
| Lai Shushen, Shan Hanrong | 2567 | 1998-06-05 | Jiangxi, China | NAS00615413 | NAS | georeferenced |
| Lai Shushen, Shan Hanrong | 4879 | 1998-06-10 | Jiangxi, China | NAS00615390 | NAS | georeferenced |
| Lai Shushen, Shan Hanrong | 5657 | 1998-06-20 | Jiangxi, China | NAS00620329 | NAS | georeferenced |
| Lai Xuewen, Cao Lan, Cheng Yankui, Zhong Weihong | 360402140611244LY | 2014-06-11 | Jiangxi, China | JXCM0002841 | JXCM | label |
| Li Enxiang, Liu Yizhen, Cai Qiying, Fang Quan, Li Wen | 15070758 | 2015-07-17 | Jiangxi, China | JJF00022517 | JJF | georeferenced |
| Li Enxiang, Liu Yizhen, Cai Qiying, Fang Quan, Li Wen | 15070907 | 2015-07-18 | Jiangxi, China | JJF00022518 | JJF | georeferenced |
| Li Enxiang, Liu Yizhen, Cai Qiying, Fang Quan, Li Wen | L2015070280 | 2015-07-18 | Jiangxi, China | JXU0015699 | JXU | georeferenced |
| Li Enxiang | L2015070319 | 2015-07-20 | Jiangxi, China | JXU0015832 | JXU | georeferenced |
| Li Juping | Lijuping0070 | 2008-09-03 | Hubei, China | 1330270 | KUN | label |
| Li Peiwu | 11039 | 1959-08-25 | Shaanxi, China | 0417983 | KUN | georeferenced |
| Li Peiwu | 9344 | 1959-05-14 | Hubei, China | 0417962 | KUN | georeferenced |
| Li Peiyuan | 10948 | 1959-08-19 | Shaanxi, China | 0140105; 0357587 | WUK | georeferenced |
| Li Peiyuan | 11039 | 1959-06-25 | Shaanxi, China | 0357404 | WUK | georeferenced |
| Li Peiyuan | 11039 | 1959-08-25 | Shaanxi, China | 140791 | WUK | georeferenced |
| Li Peiyuan | 9094 | 1959-09-03 | Shaanxi, China | 0356240 | WUK | georeferenced |
| Li Peiyuan | 9094 | 1959-09-03 | Shaanxi, China | 12000137407 | WUK | georeferenced |
| Li Peiyuan | 9344 | 1959-05-14 | Hubei, China | 0256343 | WUK | georeferenced |
| Li Peiyuan | 9609 | 1959-08-04 | Shaanxi, China | 0138593; 0356799 | WUK | georeferenced |
| Li Qiang | 1-0224 | 1986-06-18 | Shaanxi, China | 01637968 | PE | georeferenced |
| Li Qiuhe | J8112-120 | 1983-09-13 | Zhejiang, China | 1637371 | PE | georeferenced |
| Li Shuchun | — | 1950-08-26 | Anhui, China | CAF00024557 | CAF | georeferenced |
| Li Sifeng, Li Bin, Zhang Ying, Hu Hao | 17878 | 2013-08-14 | Shaanxi, China | XBGH009093; XBGH014137 | XBGH | label  label |
| Lin Feng | 84083 | 1984-07-28 | Jiangxi, China | IBK00374735 | IBK | georeferenced |
| Liu Fangxun | 10160 | 1958-07-16 | Anhui, China | HZ026014 | HHBG | georeferenced |
| Liu Fangzhao, Deng Maobin et al. | 2607 | 1956-09-11 | Jiangsu, China | 417969 | KUN | georeferenced |
| Liu Jiang | 00066 | 2012-08-05 | Shandong, China | SDF1007613 | SDFGR | georeferenced |
| Liu Li | — | 2015-07-06 | Anhui, China | ANUB002150 | ANUB | georeferenced |
| Liu Miao et al. | H40043 | 2005-07-18 | Henan, China | 1348593 | PE | georeferenced |
| Liu Qinglin | 87 | 1986-07-29 | Japan | XBGH003318 | XBGH | georeferenced |
| Liu Xiaolong | 111 | 1987-08-12 | Anhui, China | 01636358 | PE | georeferenced |
| Liu Yezhen | 14376 | 2014-08-12 | Jiangxi, China | JJF00022515; JJF00022516 | JJF | georeferenced |
| Lu Xiuli | 77 | 1983-09-13 | Zhejiang, China | FJSI021019 | FJSI | georeferenced |
| M. Chen | 519 | 1933-05-08 | Jiangsu, China | 00093016; 00093022 | PE | georeferenced |
| Ma Dandan, Shou Haiyang | ZJFC458 | 2007-07-04 | Zhejiang, China | 01878112 | PE | georeferenced |
| Ma Yuanjun | 3934 | 1978-07-19 | Hubei, China | 01636353 | PE | georeferenced |
| Mao Shaohua et al. | 140 | 1962-06-28 | Jiangsu, China | 221578 | WUK | georeferenced |
| Mao Shaohua et al. | 160 | 1962-06-28 | Jiangsu, China | 417967 | KUN | georeferenced |
| Mao Shaohua | 33 | 1962-06-23 | Jiangsu, China | IBK00174841 | IBK | georeferenced |
| Mao Zhuping | 169 | 1965-06-25 | Jiangsu, China | 0221578 | WUK | georeferenced |
| Maximowicz CJ | Maximowicz, CJ s.n. | 1863-01-01/1863-12-31 | Japan | L.2324928 | Botany | georeferenced |
| Maximowicz, -- | s.n. | 1863 | Japan | US 16608 | US | georeferenced |
| Maximowicz, C.J. | s.n. | 1863-01-01 | Japan | K000723046 | K | georeferenced |
| Maximowicz, C.J. |  | 1863 | Japan | M-0211846 / 625626 / 283288 | BSMvplantscoll | georeferenced |
| Maximowicz | — | 1863 | Japan | S-G-5219 | S | georeferenced |
| Maximowicz | — | 1863 | Japan, Nagasaki, Higo | L.203046; MEL 3499948; US 16608 | US | georeferenced |
| Mell,R.E. Plantae mellianae sinenses 656 | 656 | 1920-08-09 |  | 0170550 | WU | georeferenced |
| Miyoshi Furuse | 52555 | 1984-07 | Japan, Hiroshima, Hiba-gun, Teisyaku-kyoo | PE01569338 | PE | georeferenced |
| Mo Guixian | — | 1984-05-01 | Hunan, China | CSFI023570 | CSFI | georeferenced |
| Murata J | Murata, J 10020 | 1980-09-12 | Japan | L.2324930; WAG.1250662 | Botany | georeferenced |
| N. Eri | 100568 | 1926-06-12 | Japan, Miyazaki, Ukinjo | US 1347299 | US | georeferenced |
| Nanjing Forestry Collection Team | 9474 | 1958-07-22 | Jiangsu, China | 209177 | WUK | georeferenced |
| Nanjing Forestry Specimen | 7250 | 1905-05-11 | Jiangsu, China | HZ026020 | HHBG | georeferenced |
| Nanjing Forestry Specimen | 7800 | 1958 | Jiangsu, China | HZ026018 | HHBG | georeferenced |
| Nanjing University Collection Team | 303 | 1958-08-07 | Jiangsu, China | 0210088 | WUK | georeferenced |
| Nie Minxiang | 94277 | 1994-07-09 | Jiangxi, China | 01636351 | PE | georeferenced |
| Niu Junfeng, Tian Mo | TianXH928 | 2013-06-29 | Shaanxi, China | 1330259 | KUN | label |
| Niu Junfeng, Tian Mo | TianXH952 | 2013-08-04 | Shaanxi, China | 1330262 | KUN | label |
| Northwest University Ankang Wild Survey Team | III0027 | 1958-07-25 | Shaanxi, China | 01637259 | PE | georeferenced |
| Oldham R | Oldham, R 165 | 1862-01-01 | Japan | L 0553944 | Botany | georeferenced |
| Oldham, R. (no. 165) | — | 1862 | Japan | M-0211847 / 625627 / 283289 | BSMvplantscoll | georeferenced |
| Oldham, R. | 165 | 1862-01-01 | Japan | K000723048 | K | georeferenced |
| P. C. Tsoong | 3621 | 1936-07-25 | Anhui, China | 0051379 | WUK | georeferenced |
| Paul W. Meyer | 163 | 1981-10-02 |  | MOAR0008071 | MOAR | georeferenced |
| Paul W. Meyer | 163 | 1981-10-02 | Korea, Wolnam San, Yongin | 81-502 | MOAR | georeferenced |
| Pei Yuanrong | 006 | 1958-08 | Henan, China | 0350382 | HENU | georeferenced |
| Pei Yuanrong | 011 | 1957-08 | Henan, China | 0350373 | HENU | georeferenced |
| Pei Yuanrong | 012 | 1956-09 | Henan, China | 350383 | HENU | georeferenced |
| Pei Yuanrong | 014 | 1957-08 | Henan, China | 0350374 | HENU | georeferenced |
| Pei Yuanrong | 018 | 1956-08 | Henan, China | 0350387 | HENU | georeferenced |
| Pei Yuanrong | 101 | 1959-09 | Henan, China | 0350393 | HENU | georeferenced |
| Pei Yuanrong | 121 | 1958-09 | Henan, China | 0350389 | HENU | georeferenced |
| Pei Yuanrong | 15 | 1956-08 | Henan, China | 0350386 | HENU | georeferenced |
| Pei Yuanrong | 192 | 1956-08 | Henan, China | 0350377 | HENU | georeferenced |
| Pei Yuanrong | 4 | 1958-08 | Henan, China | 0350381 | HENU | georeferenced |
| Pei Yuanrong | 500 | 1959-06 | Henan, China | 0350380 | HENU | georeferenced |
| Pei Yuanrong | 8057 | 1905-05-12 | Henan, China | 0350372 | HENU | georeferenced |
| Pei Yuanrong | 86 | 1956-08 | Henan, China | 0350378 | HENU | georeferenced |
| Plant Resource Expedition Team | D0896 | 1984-07-15 | Henan, China | 01637957; 01637958 | PE | georeferenced |
| Qiao Yinglin | 1169 | 1959-04-25 | Shaanxi, China | 133757 | WUK | georeferenced |
| Qiu Lianqing | 4130 | 1965-06-05 | Shandong, China | 312088 | WUK | georeferenced |
| S. Hatusima | 17814 | 1955-05-14 | Japan | US 2243345 | US | georeferenced |
| S. Hatusima | 17814 | 1955-05-14 | Japan, Okinawa, Nakijin, Shoshi | US 2243345 | US | georeferenced |
| S. P. Ko | 52749 | 1933-05-28 | Guangdong, China | A00051373 | HUH | georeferenced |
| S. P. Ko | 52749 | 1933-05-28 | Guangdong, China | 00415048 | NY | georeferenced |
| S. Saito | — | 1927-06-20 | Japan, Yamaguchi, Chomonkyo, Aburun | PE01688432/TI1387 | PE/TI | georeferenced |
| S. Saito | — | 1927-06-20 | Japan, Yamaguchi, Onomori, Nagato | MO0205632/TI1387 | MO/TI | georeferenced |
| S. Saito | — | 1927-06-22 | Japan, Yamaguchi, Omijima-Otomari | PE01688437/TI1443 | PE/TI | georeferenced |
| S. Tawada | s.n. | 1966-06-21 | Japan | US 2591763A | US | georeferenced |
| Saito S | Saito, S s.n. | 1927-06-20 | Japan | L.4197492 | Botany | georeferenced |
| Saito, S. | 1387 | 1927-06-20 | Japan | 105117624 | MO | georeferenced |
| Shaanxi Wild Plant Survey Team | 278 | 1958-08-01 | Shaanxi, China | 0120384 | WUK | georeferenced |
| Shunichi Sato | 82 | 2016-05-15 | Japan | BM013850789 | BOT | label |
| Su Songwang | 662 | 1958-08-23 | Jiangsu, China | 196947 | WUK | georeferenced |
| T.N.Liou & K.M.Liou | 1195 | 1933-08-19 | Shandong, China | 9410 | WUK | georeferenced |
| T.N.Liou & P. C. Tsoong | 3024 | 1935-08-24 | Anhui, China | 0009412 | WUK | georeferenced |
| T.N.Liou | 1720 | 1929-08-19 | Shanghai, China | 93017 | PE | georeferenced |
| T.Yamazaki & H.Ohba | 529 | 1974-07-08 | Japan | 00093048 | PE | georeferenced |
| T.Yamazaki & H.Ohba | 529 | 1974-07-08 | Japan, Kyushu, Kagoshima, Hirakawa-Eboshidake | PE01217757/TI000529 | PE/TI | georeferenced |
| Tan Ceming, Dong Anmiao et al. | TanCM289 | 2010-08-11 | Jiangxi, China | 1330267; 1330268 | KUN | Label  label |
| Tan Ceming, Li Xiuzhi, Zhang Liping | 10364 | 2010-06-02 | Jiangxi, China | JJF00022511 | JJF | georeferenced |
| Tan Ceming, Yi Fabin | 05260 | 2005-06-25 | Jiangxi, China | SZG00077076 | SZG | georeferenced |
| Tan Ceming, Yi Fabin | 5260 | 2005-06-25 | Jiangxi, China | SZG00077075 | SZG | georeferenced |
| Tan Ceming, Zhang Liping and Nanchang Institute of Technology Intern Students | 8933 | 2008-06-15 | Jiangxi, China | JJF00022505; SZG00077129 | JJF/SZG | georeferenced |
| Tan Ceming, Zhu Dahai | 95501 | 1995-08-01 | Jiangxi, China | 17947 | JJF | georeferenced |
| Tan Ceming, Zhu Dahai | 95602 | 1995-08-19 | Jiangxi, China | 00017948 | JJF | georeferenced |
| Tan Ceming | 94843 | 1994-08-16 | Jiangxi, China | SN006390; SZG00048442 | SN/SZG | georeferenced |
| Tan Ceming | 95501 | 1995-08-01 | Jiangxi, China | 1636885 | PE | georeferenced |
| Tan Ceming | 95501 | 1995-08-01 | Jiangxi, China | 0452706 | KUN | georeferenced |
| Tan Ceming | 97851C | 1997-06-15 | Jiangxi, China | 00017945 | JJF | georeferenced |
| Tan Ceming | 97851C | 1997-07-20 | Jiangxi, China | SZG00077052 | SZG | georeferenced |
| Tan Zemin, Rao Linyang | 82169 | 1982-08-03 | Jiangxi, China | 17950 | JJF | georeferenced |
| Tang Gengguo | 24132 | — | Fujian, China | SWFC0006654 | SWFC | georeferenced |
| Tawada S | Tawada, S s.n. | 1966-06-21 | Japan | L.2324931 | Botany | georeferenced |
| Tian Qi, Zhang Xianquan, Zhou Xiangyu | CSYZXZ06-0187 | 2006-06-23 | Jiangxi, China | JXCM0002839 | JXCM | georeferenced |
| Tian Qi, Zhou Xiangyu, Zhang Xianquan | CSYZXZ05-0074 | 2005-09-23 | Zhejiang, China | CSH0089336 | CSH | georeferenced |
| U.J. Faurie | Faurie 1623 | 1907-08 | Korea, Republic of | FKP:84297 | A | georeferenced |
| U.J. Faurie | Faurie 2007 | 1907-07-01 | Korea, Republic of | FKP:87535 | E | georeferenced |
| U.J. Faurie | Faurie 504 | 1906-10 | Korea, Republic of | FKP:94674 | E | georeferenced |
| W.Y .Hsia | — | 1927-08-09 | Zhejiang, China | 0009403 | WUK | georeferenced |
| Walker EH | Walker, EH 8402 | 1966-06-24 | Japan | L.2324929 | Botany | georeferenced |
| Wang Huake et al. | 2004 | 2002-07-25 | Anhui, China | 01637253 | PE | georeferenced |
| Wang Jinwu | 6 | 1974-08-02 | Jiangsu, China | PEY0036595; PEY0036596 | PEY | georeferenced |
| Wang Zhaoyin, Wu Baocheng | SCSB-JS0139 | 2007-07-31 | Jiangsu, China | 1330269 | KUN | label |
| Wang Zhongli | 0004010154 | 2012-09-16 | Shandong, China | SDF1007618 | SDFGR | georeferenced |
| Wang Zhongli | 604010026 | 2012-09-16 | Shandong, China | SDF1007616 | SDFGR | georeferenced |
| Wang Zuobin | 18299 | 1958-05-31 | Shaanxi, China | 0098881 | WUK | georeferenced |
| Wilson, E.H. | 1515 | 1907-07-27 | Jiangxi, China | K004542048 | K | georeferenced |
| Wilson, E.H. | 3389 | 1907-07-01 | Western Hubei, China | K000723043 | K | georeferenced |
| Wilson, Ernest Henry | 1515 | 1907-07-27 | Jiangxi, China | E00275330（Type Specimen） | E | georeferenced |
| Workstation Staff | 3013 | 1951-07-23 | Jiangsu, China | 417972 | KUN | georeferenced |
| Wu Jinqing | 9444 | 2006-07-30 | Hubei, China | HIB0177748 | HIB | georeferenced |
| Wu Wenxiang | 3739 | 1958-07-25 | Jiangsu, China | 1637378 | PE | georeferenced |
| Wu Wenxiang | 4336 | 1958-08-09 | Jiangsu, China | 1637381 | PE | georeferenced |
| Wu Wenxiang | 4596 | 1958-08-19 | Jiangsu, China | 209822 | WUK | georeferenced |
| Wu Wenxiang | 4614 | 1958-08-19 | Jiangsu, China | 1637382 | PE | georeferenced |
| Wu Wenxiang | 4614 | 1958-08-29 | Jiangsu, China | 196916 | WUK | georeferenced |
| Wu Wenxiang | 5988 | 1967-07-28 | Jiangsu, China | NAS00583453; NAS00586704 | NAS | georeferenced |
| Wu Wenxiang | s.n. | 1958-08-16 | Jiangsu, China | 1637377 | PE | georeferenced |
| Wu Xudong et al. | L9113388 | 1992-01-25 | Zhejiang, China | ZMNH0046898 | ZM | georeferenced |
| Wu Yan | 012 | 2015-07-06 | Anhui, China | ANUB002273 | ANUB | georeferenced |
| Xin Hua | 25 | 2012-08-17 | Shandong, China | SDF1007623 | SDFGR | georeferenced |
| Xin Hua | 9 | 2012-09-22 | Shandong, China | SDF1007615 | SDFGR | georeferenced |
| Xin Xiaowei, Bu Ruilan, Shi Liang | XINGJ112 | 2022-08-24 | Shandong, China | 1596745 | KUN | label |
| Xing Jiqing | 10993 | 1959-08-09 | Hubei, China | 0383845; 0128866 | WUK | georeferenced |
| Xing Jiqing | 11326 | 1959-08-15 | Hubei, China | 0129394 | WUK | georeferenced |
| Xing Jiqing | 11396 | 1959-08-15 | Hubei, China | 0128966; 0384231 | WUK | georeferenced |
| Xing Jiqing | 15827 | 1959-08-24 | Hubei, China | 0131268 | WUK | georeferenced |
| Xing Jiqing | 15948 | 1959-08-24 | Hubei, China | 0131095 | WUK | georeferenced |
| Xing Jiqing | 16024 | 1959-08-24 | Hubei, China | 0387127; 0417963 | KUN/WUK | georeferenced |
| Xing Jiqing | 16145 | 1959-08-24 | Hubei, China | 131134; 417965 | KUN/WUK | georeferenced |
| Xing Jiqing | 16840 | 1959-08-28 | Hubei, China | 386659 | WUK | georeferenced |
| Xing Jiqing | 17190 | 1959-08-29 | Hubei, China | 0131158 | WUK | georeferenced |
| Xing Jiqing | 17200 | 1959-08-27 | Hubei, China | 0417960; 0387032 | KUN/WUK | georeferenced |
| Xing Jiqing | 17850 | 1959-08-28 | Hubei, China | 0130976; 0387168 | WUK | georeferenced |
| Xiong Yuning, Chu Xiaofang, Gu Zixia et al. | 448 | 2018-06-05 | Anhui, China | NAS00620295 | NAS | label |
| Xiong Yuning, Chu Xiaofang, Gu Zixia et al. | 448 | 2018-06-05 | Anhui, China, Shitai, Dayan | NAS00620296 | NAS | label |
| Xiong Yuning, Jiang Ruping, Zhang Jianhua | 1185 | 2017-09-13 | Anhui, China | NAS00626274; NAS00626275; NAS00626276 | NAS | georeferenced |
| Xiong Yuning, Ma Zhenxiu, Wu Baocheng | 5240 | 2012-07-19 | Jiangsu, China | NAS00572371 | NAS | georeferenced |
| Xu Honggui et al. | 171 | 1958-08-18 | Jiangsu, China | 00093018 | PE | georeferenced |
| Xu Zenglai, Wu Baocheng | 0842 | 2011-08-21 | Jiangsu, China | NAS00620224 | NAS | georeferenced |
| Xu Zenglai, Wu Baocheng | 104 | 2011-08-16 | Jiangsu, China | NAS00620287 | NAS | georeferenced |
| Xu Zenglai, Wu Baocheng | 42 | 2011-08-15 | Jiangsu, China | NAS00620285 | NAS | georeferenced |
| Xu Zenglai, Wu Baocheng | 842 | 2011-08-21 | Jiangsu, China | NAS00620225 | NAS | georeferenced |
| Xu Zenglai | 842 | — | Jiangsu, China | NAS00620242 | NAS | georeferenced |
| Xunyang Sub-team | 0425 | 1959-07-02 | Shaanxi, China | 01637255; 01637256 | PE | georeferenced |
| Xunyang Sub-team | 425 | 1959-07-02 | Shaanxi, China | 01637257 | PE | georeferenced |
| Xunyang Team | 0095 | 1959-07-01 | Shaanxi, China | 01637260 | PE | georeferenced |
| Xunyang Team | 95 | 1959-07-01 | Shaanxi, China | 01637261 | PE | georeferenced |
| Yang Chen | 005 | 1987-08 | Henan, China | 0350390 | HENU | georeferenced |
| Yang Chen | 007 | 1987-08 | Henan, China | 0350391 | HENU | georeferenced |
| Yang Chen | 367 | 1985-07 | Henan, China | 0350385 | HENU | georeferenced |
| Yang Shiji, Deng Zhi, Yue Junsan | 471 | 1953-06-03 | Jiangsu, China | 0110914 | WUK | georeferenced |
| Yang Shixuan | 526 | 1985-08-17 | Hubei, China | IBK00352018 | IBK | georeferenced |
| Yang Yongjie | 064 | 2012-09-11 | Shandong, China | SDF1007627 | SDFGR | georeferenced |
| Yao Gan et al. | 79270 | 1980-08-29 | Zhejiang, China | FJSI021017 | FJSI | georeferenced |
| Yaopu Team | 002 | 1961-08-13 | Jiangsu, China | NAS00578107 | NAS | georeferenced |
| Ye Dexian | 1241 | 1958-07-29 | Jiangsu, China | 0209856 | WUK | georeferenced |
| Ye Dexian | 1381 | 1958-08-04 | Heilongjiang, China | 0196919 | WUK | georeferenced |
| Ye Dexian | 1381 | 1958-08-04 | Shanghai, China | HZ026010 | HHBG | georeferenced |
| Ye Dexian | 1543 | 1958-08-08 | Jiangsu, China | 0210090 | WUK | georeferenced |
| Ye Dexian | 1881 | 1958-08-04 | Jiangsu, China | 0210089 | WUK | georeferenced |
| Ye Huagu, Zeng Feiyan et al. | 23917 | 2019-06-15 | Henan, China | 856464 | IBSC | label |
| Ye Huagu, Zeng Feiyan et al. | 24444 | 2019-09-16 | Henan, China | 856540 | IBSC | label |
| Ye Huagu, Zeng Feiyan et al. | LXP10-3635 | 2014-05-19 | Jiangxi, China | 830041 | IBSC | georeferenced |
| Yi Guihua, Zhang Liping | 9594 | 2009-07-02 | Jiangxi, China | JJF00022509; JJF00022510; SZG00077058 | JJF/SZG | georeferenced |
| Yi Guihua | 16061334 | 2016-06-30 | Jiangxi, China | JJF00022519; JJF00022520 | JJF | georeferenced |
| Anonymous | K30698 | 2012-05-13 | Japan | KAG023446 | KAG | label |
| Anonymous | — | 2017-06-20 | Japan | KAG019217 | KAG | georeferenced |
| Anonymous | — | 2020-08-04 | Japan Amami Islands Amami-oshima Island Kagoshima Oshima-gu Tatugo-cho | KAG023984 | KAG | georeferenced |
| Anonymous | — | 2020-08-07 | Japan | KAG024857 | KAG | georeferenced |
| Anonymous | — | — | Japan | GA183431 | GA | label |
| Anonymous | — | — | Korea, Republic of | GA183432 | GA | georeferenced |
| Yuan Fayin | 11134 | 1981-08-17 | Jiangsu, China | IBK00271914 | IBK | georeferenced |
| Zhan Xuanhuai, Peng Yansong et al. | LXP0072 | 2013-09-14 | Jiangxi, China | LBG00138965 | LBG | georeferenced |
| Zhan Xuanhuai, Peng Yansong et al. | LXP0072 | 2013-09-14 | Jiangxi, China | LBG00138966 | LBG | georeferenced |
| Zhan Xuanhuai, Peng Yansong et al. | LXP0164 | 2013-09-15 | Jiangxi, China | LBG00130205 | LBG | georeferenced |
| Zhan Xuanhuai, Peng Yansong et al. | LXP0164 | 2013-09-15 | Jiangxi, China | LBG00130204 | LBG | georeferenced |
| Zhan Xuanhuai, Peng Yansong et al. | LXP1731 | 2014-07-02 | Hubei, China | LBG00132622 | LBG | georeferenced |
| Zhan Xuanhuai, Peng Yansong et al. | LXP1732 | 2014-07-02 | Hubei, China | LBG00132623 | LBG | georeferenced |
| Zhang Jihua | 2327 | 2016-07-02 | Jiangxi, China | JJF00022521; JJF00022522 | JJF | georeferenced |
| Zhang Jihua | Tancm2943 | 2016-07-08 | Jiangxi, China | JJF00022523; JJF00022524 | JJF | georeferenced |
| Zhang Ping | 0004120058 | 2012-08-01 | Shandong, China | SDF1007612 | SDFGR | georeferenced |
| Zhang Ping | 0611040039 | 2012-06-25 | Shandong, China | SDF1007619 | SDFGR | georeferenced |
| Zhang Siyu, Zhu Kerun, Shao Jianwen | ZSY0150 | 2020-08-18 | Anhui, China | HIB0205617; HIB0205618 | HIB | label  label |
| Zhang, Xu | 0041 | 1989-08-17 | Zhejiang, China | ZMNH0046875 | ZM | georeferenced |
| Zhao Xianhua | 1183 | 1978-09 | Henan, China | 350369 | HENU | georeferenced |
| Zhao Xianhua | 1184 | 1978-09 | Henan, China | 350370 | HENU | georeferenced |
| Zhao Xianhua | 134 | 1958-07-01 | Henan, China | 350371 | HENU | georeferenced |
| Zhao Xianhua | 78103 | 1980-06 | Henan, China | 350367 | HENU | georeferenced |
| Zhejiang Plant Resource Census Team | 28960 | 1959-08-23 | Zhejiang, China | HZ025993 | HHBG | georeferenced |
| Zheng Zhong | 145 | 1963-06-20 | Henan, China | 0311235 | WUK | georeferenced |
| Zheng Zhong | 206 | 1963-06-20 | Henan, China | 0311286 | WUK | georeferenced |
| Zhou Hongbin | 2626 | 1958-08-08 | Jiangsu, China | 01637380; 0197399 | PE/WUK | georeferenced |
| Zhou Hongbin | 2916 | 1958-08-14 | Jiangsu, China | 0209578 | WUK | georeferenced |
| Zhou Yifeng, Dong Xiaoyu, Xiong Yuning, Wang Xiang | 20191133 | 2019-09-15 | Zhejiang, China | NAS00636216; NAS00636217 | NAS | georeferenced |
| Zhou Zhongze | 0009 | 2002-07-10 | Anhui, China | 01637252 | PE | georeferenced |
| Zhu Changshan, Li Shijin, Hou Xueliang, Zhu Shixin, Li Jiamei | 1308101 | 2013-08-10 | Henan, China | AU067074 | AU | georeferenced |
| Zhu Changshan, Li Shijin,Hou Xueliang, Zhu Shixin, Li Jiamei, Zhang Yunxia, Li Jingzhao, Yang Wenguang, Zhang Ruopeng | 150805036 | 2015-08-05 | Henan, China | AU064307 | AU | georeferenced |
| Zhu Heqing | 549 | — | Zhejiang, China | HZ026003 | HHBG | georeferenced |
| Zou Wenxiang | 4614 | 1958-08-19 | Jiangsu, China | 209665 | WUK | georeferenced |
| s.coll. | s.n. | — | Korea, Republic of | K004542096 | K | georeferenced |
| Anonymous | 387 | 1988-07-29 | Japan, Kyoto Botanical Garden | XBGH003318 | XBGH | georeferenced |
| Anonymous | — | 1902-07-13 | Japan, Botanical Garden | 00093044 | PE | georeferenced |
| Anonymous | — | 1910-08-14 | Japan, Tottori | US 1155602 | US | georeferenced |

Note: The Coordinate source column indicates how the mapping coordinates (Figure 2) were obtained from each specimen (‘label’ = coordinates printed on the label; ‘georeferenced’ = coordinates inferred from the locality on the label). Coordinates of the newly collected populations in Table 1 were recorded by GPS.

**Supplementary Table S3.** Type specimens examined for PCA and morphological comparison in this study.

| **Species name** | **Barcode information** |
| --- | --- |
| *Berchemia congesta* S.Moore. | K000723049 |
| *Rhamnella obovalis* C.K.Schneid. | A 00032772 |
| *Rhamnella obovalis* C.K.Schneid. | US 00094390 |
| *Rhamnella obovalis* C.K.Schneid. | E00275330 |
| *Rhamnella obovalis* C.K.Schneid. | E00275331 |
| *Rhamnella caudata* Merr. | PE00023560 |
| *Rhamnella caudata* Merr. | PE00023559 |
| *Rhamnella caudata* Merr. | A00051373 |
| *Rhamnella japonica* Miq. | L 0553944 |
| *Rhamnella franguloides* var. *franguloides* | K000723046 |
| *Rhamnella franguloides* var. *franguloides* | S-G-5219 |

**Table S4.** Individual-level sequence information for all newly collected samples.

| **Cluster or outgroup** | **GenBank accession No.** | **Voucher number** | **Locality** |
| --- | --- | --- | --- |
| Cluster A | PZ530784 | 2025PL01 | Pingli, Shaanxi, China |
| Cluster A | PZ530785 | 2025XD01 | Xiangxidong, Shaanxi, China |
| Cluster A | PZ530787 | 2025YJ01, 2025YJ02, 2025YJ03 | Yuhuangjian, Henan, China |
| Cluster A | PZ530788 | 2025NJ01, 2025NJ02, 2025ZC01, 2025ZC02, 2025FX01, 2025FX02, 2025FX03 | Nanjing, Jiangsu, China  Zoucheng, Shandong, China  Fangxian, Hubei, China |
| Cluster A | PZ530786 | 2025FS01, 2025FS02, 2025YJ04, 2025PL02 | Fanguangsi, Zhejiang, China  Yuhuangjian, Henan, China  Pingli, Shaanxi, China |
| Cluster B | PZ530789 | 2025RY01, 2025RY02, 2025RY04, 2025RY06, 2025RY07, 2025RY09, 2025RY11, 2025RY13 | Ruyuan, Guangdong,China |
| Cluster B | PZ530790 | 2025RY03, 2025RY08, 2025RY12 | Ruyuan, Guangdong,China |
| Cluster B | PZ530791 | 2025RY05, 2025RY10 | Ruyuan, Guangdong,China |
| *R. martini* | PZ530792 | 2025XS01 | Xishan, Yunnan, China |
| *R. martini* | PZ530793 | 2025XS02 | Xishan, Yunnan, China |
| *R. julianae* | PZ841209 | 2026WC01 | Wenchuan, Sichuan, China |
| *R. julianae* | PZ841210 | 2026WX01 | Wuxi, Chongqing, China |
| *R. julianae* | PZ841211 | 2026MY01 | Muyu, Hubei, China |

**Figure S1.** **Diagram of leaf morphological traits** in Tables 2 and 3**.**

Fig. S1 Diagram of leaf morphological traits and their measurement methods.

Trait Definitions: a. Leaf length (cm); b. Leaf width (cm); c. Leaf length-width ratio; d. Leaf petiole length (cm); e. Leaf apex length (cm); f. Leaf lateral vein number per side; g. a/f.
